# CRISPR-Cas9-mediated functional dissection of the *foxc1* genomic region in zebrafish identifies critical conserved *cis*-regulatory elements

**DOI:** 10.1186/s40246-022-00423-x

**Published:** 2022-10-25

**Authors:** Jesús-José Ferre-Fernández, Sanaa Muheisen, Samuel Thompson, Elena V. Semina

**Affiliations:** 1 Department of Pediatrics and Children’s Research Institute, Medical College of Wisconsin and Children’s Hospital of Wisconsin, Milwaukee, WI 53226 USA; 2 Department of Ophthalmology and Visual Sciences, Medical College of Wisconsin, Milwaukee, WI 53226 USA; 3 Department of Cell Biology, Neurobiology and Anatomy, Medical College of Wisconsin, Milwaukee, WI 53226 USA

## Abstract

*FOXC1* encodes a forkhead-domain transcription factor associated with several ocular disorders. Correct *FOXC1* dosage is critical to normal development, yet the mechanisms controlling its expression remain unknown. Together with *FOXQ1* and *FOXF2*, *FOXC1* is part of a cluster of *FOX* genes conserved in vertebrates. CRISPR-Cas9-mediated dissection of genomic sequences surrounding two zebrafish orthologs of *FOXC1* was performed. This included five zebrafish–human conserved regions, three downstream of *foxc1a* and two remotely upstream of *foxf2a/foxc1a* or *foxf2b/foxc1b* clusters, as well as two intergenic regions between *foxc1a/b* and *foxf2a/b* lacking sequence conservation but positionally corresponding to the area encompassing a previously reported glaucoma-associated SNP in humans. Removal of downstream sequences altered *foxc1a* expression; moreover, zebrafish carrying deletions of two or three downstream elements demonstrated abnormal phenotypes including enlargement of the anterior chamber of the eye reminiscent of human congenital glaucoma. Deletions of distant upstream conserved elements influenced the expression of *foxf2a/b* or *foxq1a/b* but not *foxc1a/b* within each cluster. Removal of either intergenic sequence reduced *foxc1a* or *foxc1b* expression during late development, suggesting a role in transcriptional regulation despite the lack of conservation at the nucleotide level. Further studies of the identified regions in human patients may explain additional individuals with developmental ocular disorders.

**Supplementary Information:**

The online version contains supplementary material available at 10.1186/s40246-022-00423-x.

## Introduction

*FOXC1* encodes a transcription factor that binds regulatory DNA elements of its direct targets via a winged helix forkhead domain [1]. Together with *FOXQ1* and *FOXF2*, *FOXC1* is part of a conserved block of *FOX* transcription factor genes on human chromosome 6. *FOXC1* is involved in the development of the anterior segment of the eye and other organs [2]. The initial *FOXC1*-related phenotype was identified as Axenfeld–Rieger Syndrome Type III (ARS, OMIM #602482), which involves posterior embryotoxon, iris hypoplasia, irido-corneal adhesions and ~ 50% chance to develop glaucoma [3]. Later, *FOXC1* mutations were discovered in patients with aniridia, Peters anomaly and primary congenital glaucoma (PCG) [4–6]. Patients with mutations in *FOXC1* often have additional non-ocular anomalies, such as heart defects, craniofacial dysmorphisms, hearing loss, skeletal anomalies (hip dysplasia or scoliosis), feeding issues, dental enamel hypoplasia, hypotonia/delay, and white matter lesions in the brain [7–11]. Mutations in *FOXC1* explain a high proportion of cases affected with ARS and related disorders; however, there is still a considerable number of patients with an unknown genetic cause [8].

The expression of developmental genes is finely controlled by their regulatory elements which are often evolutionarily conserved [12]. Mutations in regulatory elements have been implicated in eye and other developmental disorders [4, 13–16]. Moreover, genome-wide association studies (GWAS) indicated that the majority of disease-associated loci lie in noncoding regions of the genome [17]. Specific to *FOXC1*, a GWAS study discovered a SNP at ~ 61.7 kb 5′ of *FOXC1* (rs2745572[A]) that was significantly associated with primary open-angle glaucoma (POAG) and vertical cup-to-disk ratio, an important endophenotype for glaucoma [18, 19]. However, the regulatory elements of *FOXC1* are still unknown.

Zebrafish has proved to be a robust animal model for the study of genes involved in embryonic development [12, 20]. There are two genes orthologous to human *FOXC1* in zebrafish, *foxc1a* on chromosome 2 and *foxc1b* on chromosome 20 [21]. These two genes show high conservation at the protein level with 66% (Foxc1a) and 55% (Foxc1b) identity to human FOXC1 [21]. Moreover, the human and zebrafish genes are located within blocks of conserved synteny. Both *foxc1* genes are expressed in developing zebrafish embryos in overlapping but distinct patterns [22]. Studies of knockout lines for *foxc1a* and *foxc1b* identified embryonic lethality, altered somitogenesis, cardiac anomalies/heart edema, and facial cartilage defects for the *foxc1a* knockout (KO), and no visible phenotype for the *foxc1b* KO [23–26]. Our group characterized the eye phenotype of *foxc1a* KO (*mw711*) and *foxc1* double-KO lines and showed that both are similarly affected with major ocular defects overlapping ARS [26].

In order to discover and characterize regulatory elements of *FOXC1*, we performed various analyses to identify candidate regulatory regions and then used CRISPR-Cas9 technology to delete those regions in zebrafish followed by in vivo evaluations of the resultant lines. In total, five human noncoding regions corresponding to seven regions in zebrafish were examined and found to have variable effects on the expression of *foxc1* or surrounding genes.

## Results

### Identification of candidate regulatory elements in the *FOXC1* genomic region

To identify candidate regulatory elements, we performed a multispecies comparison of *FOXC1/foxc1* genomic sequences, with a focus on regions conserved between human and zebrafish. The examined area included genomic sequences of human chromosome 6 starting at ~ 1.6-Mb upstream of *FOXC1* and ending ~ 1-Mb downstream of this gene, which corresponds to ~ 213 kb and ~ 148 kb upstream and ~ 262 kb and ~ 142 kb downstream of zebrafish *foxc1a* and *foxc1b,* respectively*.* In humans, the studied region encompasses *DUSP22*, *IRF4*, *EXOC2*, *HUS1B*, *FOXQ1*, *FOXF2*, *FOXC1*, *GMDS*, 7 pseudogenes, 21 long noncoding RNA genes (18 uncharacterized), 5 long intergenic non-protein coding RNAs genes and 1 microRNA gene. In zebrafish, the chromosome 2 region contains *dusp22b, irf4a, foxq1a, foxf2a,* and *foxc1a* but not *exoc2, hus1b* and *gmds*, while the chromosome 20 region contains *irf4b, exoc2, foxq1b, foxf2b, foxc1b,* and *gmds* but not *dusp22* or *hus1b*; there was no evidence for the presence of pseudogenes, noncoding, or microRNA genes on any of the zebrafish chromosomes.

**Fig. 1:**
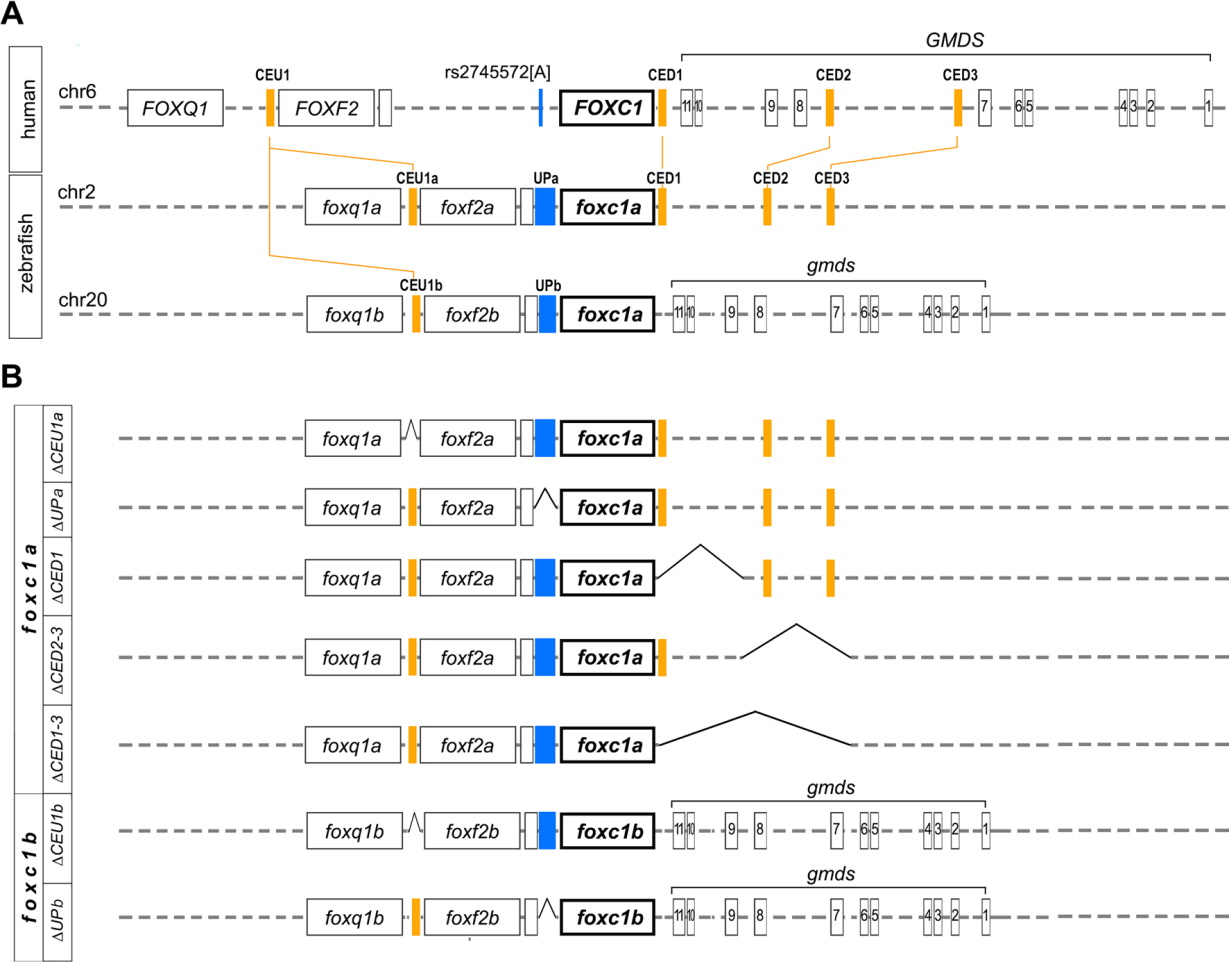
Schematic of human *FOXC1,* zebrafish *foxc1a* and *foxc1b* loci, and developed zebrafish lines. **A** Schematic drawing of human chromosome 6 aligned with zebrafish chromosomes 2 and 20 showing the positions of the identified conserved elements (filled orange boxes labeled at the top). Exons are indicated with labeled black boxes, while intergenic and intronic regions are shown with dotted lines. Position of rs2745572[A], an SNP associated with primary open-angle glaucoma, is shown in blue on human chromosome 6 and corresponding intergenic regions on zebrafish chromosomes 2 and 20 are marked with blue rectangles. **B** Schematic drawing showing generated lines and corresponding genomic deletions (sequence gaps are indicated)

This analysis identified four conserved regions in humans, one distant upstream and three downstream of *FOXC1* (Fig. 1A), that corresponded to five zebrafish regions, two distant upstream of *foxc1a* or *foxc1b* and three downstream of *foxc1a*. The conserved element upstream of *FOXC1,* named Conserved Element Upstream 1 (CEU1), is located 221 kb upstream of *FOXC1* (1.57 kb upstream of *FOXF2* and 73 kb downstream of *FOXQ1*). Sequences with high homology to CEU1 were identified on both zebrafish chromosomes 2 (CEU1a) and 20 (CEU1b), upstream of *FOXF2* orthologs *foxf2a* and *foxf2b*, respectively (Fig. 1A). The zebrafish CEU1a element is located 519-bp upstream of *foxf2a* and spans 78-bp with 78% identity with humans. The zebrafish CEU1b element is located 565-bp upstream of *foxf2b* and spans 160-bp with 69% identity with humans (Table 1; Additional file 1: Fig. S1). For the three conserved elements downstream of human *FOXC1*, the first one, named Conserved Element Downstream 1 (CED1), is situated in the intergenic region between *FOXC1* and *GMDS* at 2.9 kb from *FOXC1* (Fig. 1A), while the other two elements, CED2 and CED3, are located between exons 7 and 8 of *GMDS* (NM_001500.4) at 194.4 kb and 290 kb from *FOXC1*, respectively (Fig. 1A). All three downstream elements had homologous sequences on zebrafish chromosome 2 (*foxc1a*) but not chromosome 20 (*foxc1b*): in zebrafish, the CED1, CED2 and CED3 regions are positioned 2.5, 109.3 and 151 kb downstream of *foxc1a* and span 99-bp (76% identity), 67-bp (84% identity) and 140-bp (81% identity), respectively (Table 1; Additional file 1: Fig. S1). Review of the Encyclopedia of DNA Elements (ENCODE) [27] showed that all four human conserved regions, CEU1 and CED1-3, co-localize with predicted *cis*-regulatory elements (Table 1 and Additional file 1: Fig. S1). Also, CED1 overlaps with a previously predicted (using EnhancerFinder) enhancer region in the *FOXC1*-*GMDS* block [28].

**Table 1:** Summary of conserved elements in human *FOXC1* and zebrafish *foxc1a/b* genomic regions

Conserved element	Human coordinates (hg38)	Corresponding ENCODE cCREs*	Zebrafish coordinates (GRCzZ11)	Length of alignment (bp)	% of identity	Distance to next human gene	Distance to next zebrafish gene
CEU1	chr6:1387821–1387980	EH38E2439030 (chr6:1387819–1388167)	CEU1a chr2:716946–717020	78	78	1.57 Kb upstream of *FOXF2*	519 bp upstream of *foxf2a*
			CEU1b chr20:26689284–26689441	160	69		565 bp upstream of *foxf2b*
CED1	chr6:1616932–1617028	EH38E2439207 (chr6:1616739–1617072)	chr2:684320–684414	99	76	2.9 Kb downstream of *FOXC1*	2.5 Kb downstream of *foxc1a*
CED2	chr6:1778296–1778362	EH38E2439358 (chr6:1777933–1778281)	chr2:577491–577557	67	84	164.4 Kb downstream of *FOXC1*	109.3 Kb downstream of *foxc1a*
CED3	chr6:1903895–1904034	EH38E2439473 (chr6:1903759–1904109)	chr2:535659–535798	140	81	290 Kb downstream of *FOXC1*	151 Kb downstream of *foxc1a*

^*^cCREs = candidate *cis*-regulatory elements; cCREs that overlap or are immediately adjacent to the conserved elements are listed

Literature review additionally revealed a SNP, rs2745572[A], associated with primary open-angle glaucoma (POAG) and increased vertical cup-to-disk ratio (a glaucoma endophenotype) [18, 19], located in the intergenic region between *FOXC1* and *FOXF2* at ~ 61.7 kb upstream of *FOXC1* and ~ 152.6 kb downstream of *FOXF2*. BLAST-based comparisons of the human intergenic sequence to corresponding genomic regions between zebrafish *foxc1a/b* and *foxf2a/b* failed to identify any conserved elements in these regions.

However, the reported association possibly indicates the presence of a regulatory sequence(s) in the intergenic region between *FOXC1* and *FOXF2*.

### Generation of deletion lines in zebrafish

A CRISPR-Cas9 genome editing system was utilized to generate various zebrafish lines carrying deletions of the identified candidate regulatory regions. For the distant upstream conserved element of *FOXC1/foxc1a/foxc1b*, two different deletion lines were generated: the first line, named *foxc1a*^*∆CEU1a*^, carries a 1226-bp deletion encompassing CEU1a located upstream of *foxf2a*, while the second line, *foxc1b*^*∆CEU1b*^, carries a 779-bp deletion including CEU1b located upstream of *foxf2b* (Fig. 1B; Additional file 1: Table S1, Fig. S2A, F). In order to test upstream sequences that are positionally homologous to the human region containing POAG-associated SNP rs2745572 (but lacking any clear sequence conservation), additional lines *foxc1a*^*∆UPa*^ and *foxc1b*^*∆UPb*^, were generated carrying deletions of the entire intergenic regions between *foxf2a* and *foxc1a* (20,671-bp) or foxf2b and *foxc1b* (7726-bp), respectively, excluding the ~ 3 kb fragments immediately upstream of *foxc1a* or *b*, to ensure retainment of all promoter sequences (Fig. 1B; Additional file 1: Table S1, Fig. S2B, G).

For the conserved elements downstream of *FOXC1/foxc1a*, three different deletion lines were generated: the first line, *foxc1a*^*∆CED1*^, carries a 69,072 kb deletion containing CED1 only; the second line, *foxc1a*^*∆CED2−3*^, carries an 82,715 kb deletion including CED2 and CED3; while the third line, *foxc1a*^*∆CED1−3*^, carries a 151,989-bp deletion encompassing all three conserved elements (CED1, CED2 and CED3) downstream of *foxc1a*; (Fig. 1B; Additional file 1: Table S1, Fig. S2C–E).

### Analysis of lines carrying deletions of upstream regions of *foxc1a/b*

**Fig. 2:**
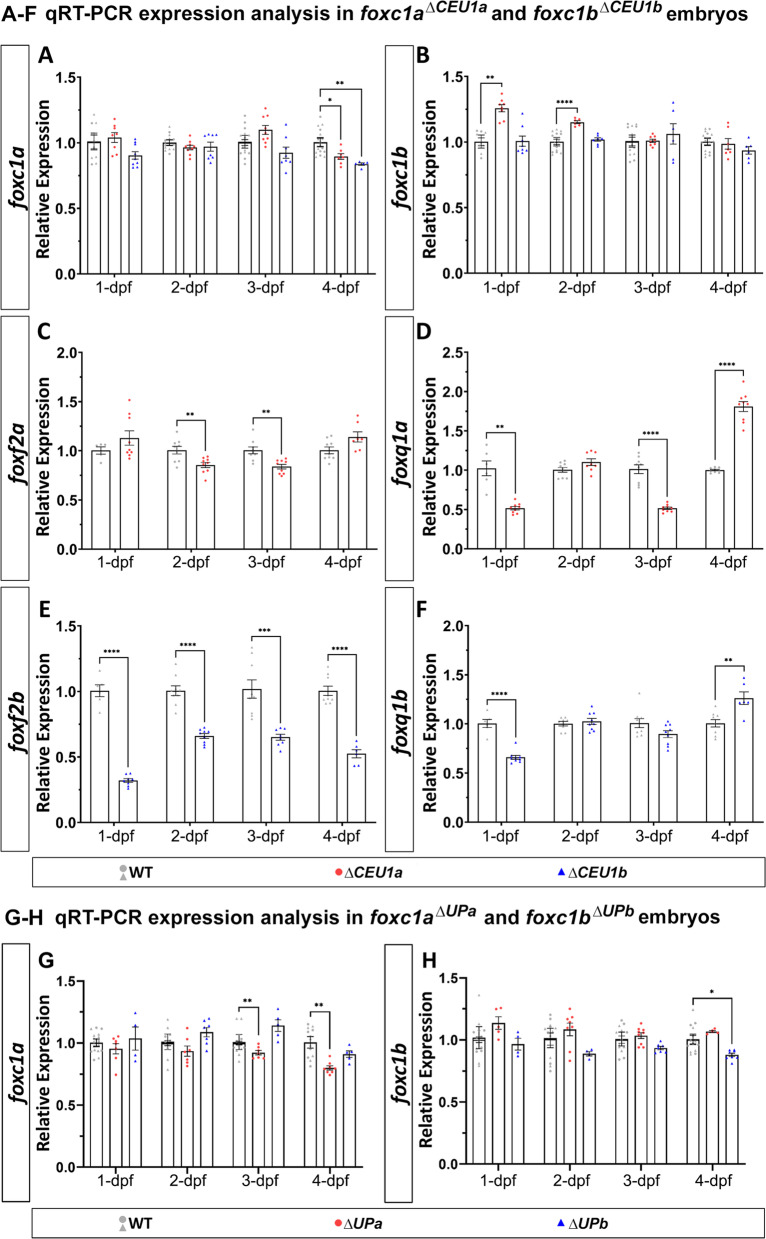
Changes in gene expression in mutants carrying deletions of upstream regions. **A**–**F** qRT-PCR relative expression of *foxc1a* (**A**) and *foxc1b* (**B**), *foxf2a* (**C**) and *foxq1a* (**D**) and *foxf2b* (**E**) and *foxq1b* (**F**) in 1-, 2-, 3- and 4-dpf wild-type, *foxc1a*^*∆CEU1a*^ and/or *foxc1b*^*∆CEU1b*^ homozygous zebrafish embryos (whole bodies). **G**, **H** qRT-PCR relative expression of *foxc1a* (**G**) and *foxc1b* (H) in 1-, 2-, 3- and 4-dpf wild-type, *foxc1a*^*∆UPa*^ and *foxc1b*^*∆UPb*^ homozygous embryos. β-actin (*actb1*) was used as the reference transcript in all experiments. *: *p* < 0.05; **: *p* < 0.01; ***: *p* < 0.001; ****: *p* < 0.0001

Careful examination of *foxc1a*^*∆CEU1a*^ and *foxc1b*^*∆CEU1b*^ lines carrying deletions of distant upstream regions did not identify any consistent phenotype in homozygous or heterozygous embryos or adults. To further evaluate their possible regulatory function, qRT-PCR analysis of *foxc1a*, *foxc1b*, *foxf2a*/*b* and *foxq1a/b* transcripts in wild-type and mutant embryos was performed. This analysis identified various mild effects on *foxc1a* and *foxc1b* expression (Fig. 2A, B), while significant changes in expression levels of *foxf2a*/b and *foxq1a/b* were observed in corresponding homozygous embryos (Fig. 2C–F). These results indicate that the distant upstream conserved elements are primarily involved in the regulation of the nearby *foxf2* and *foxq1* genes rather than *foxc1*.

Examinations of the *foxc1a*^*∆UPa*^ and *foxc1b*^*∆UPb*^ lines carrying deletions of the intergenic regions between the *foxc1a* and *foxf2a* or *foxc1b* and *foxf2b* (excluding the ~ 3 kb fragments immediately upstream of *foxc1a/b*) identified no visible phenotype in either homozygous or heterozygous animals. We next examined the expression levels of *foxc1a* and *foxc1b* in wild-type and mutant embryos at different developmental stages. A mild but statistically significant decrease in *foxc1a* or *foxc1b* levels at later stages of development was observed in the respective lines: to 0.92 and 0.8 at 3- and 4-dpf (days post-fertilization) for *foxc1a* in *foxc1a*^*∆UPa*^ embryos (Fig. 2G) and to 0.88 at 4-dpf for *foxc1b* in *foxc1b*^*∆UPb*^ mutants (Fig. 2H). These data suggest that each intergenic region may contain elements contributing to the proper expression of *foxc1a* and *foxc1b* at later stages of development.

### Analysis of lines carrying deletions of downstream conserved regions of *foxc1a*

**Fig. 3:**
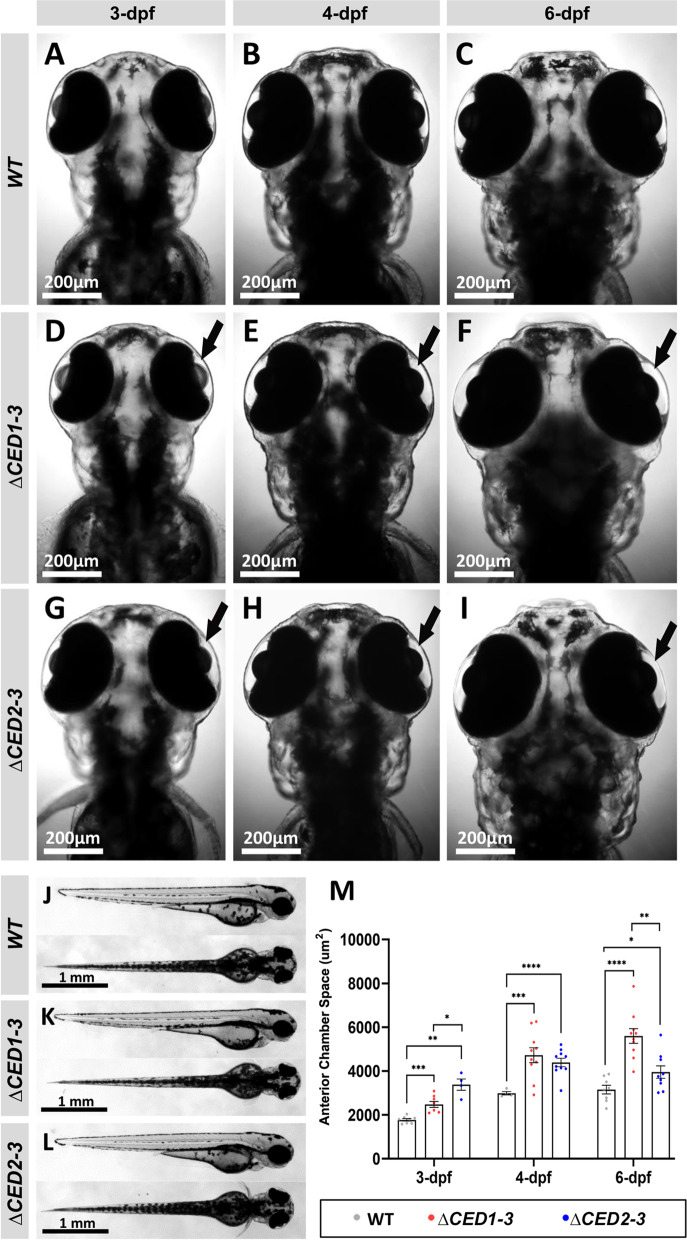
Phenotypic analysis of zebrafish mutants carrying deletions of downstream elements. **A**–**I** Dorsal images of the head region of 3-, 4- and 6-dpf wild-type (WT) (**A**–**C**), *foxc1a*^*∆CED1−3*^ (**D**–**F**) and *foxc1a*^*∆CED2−3*^ (**G**–**I**) homozygous zebrafish embryos. Both mutant lines showed the enlargement of the anterior chamber of the eye that was first noticeable at 3-dpf and became more pronounced by 6-dpf (black arrows in **D**–**I**). **J**–**L** Lateral and dorsal views of the 3-dpf wild-type (**J**), *foxc1a*^*∆CED1−3*^ (**K**) and *foxc1a*^*∆CED2−3*^ (**L**) homozygous zebrafish embryos. Please note no obvious morphological changes (aside from ocular defects presented in **A**–**I**) in mutant embryos. **M** Comparison of the anterior chamber area in wild-type and mutant embryos at 3-, 4-, and 6-dpf. *: *p* < 0.05; **: *p* < 0.01; ***: *p* < 0.001; ****: *p* < 0.0001

**Fig. 4:**
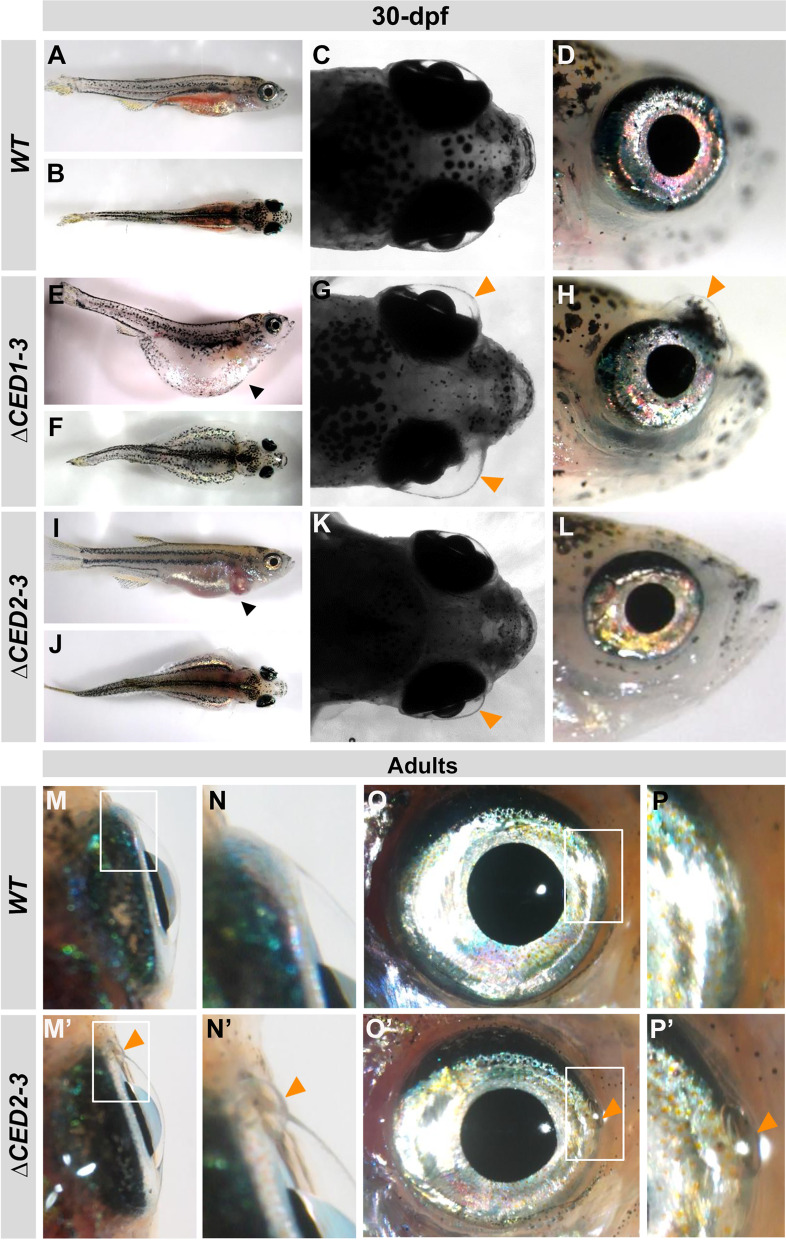
Developmental defects in juvenile and adult *foxc1a*^*∆CED1−3*^ and *foxc1a*^*∆CED2−3*^ mutants. **A**–**L** Lateral and dorsal whole body and head images of 30-dpf wild-type (**A**–**D**), *foxc1a*^*∆CED1−3*^ (**E**–**H**) and *foxc1a*^*∆CED2−3*^ (**I**–**L**) homozygous zebrafish embryos. Please note general swelling, including abdominal and heart edema (black arrowheads), in mutant embryos (**E**, **I**) as well as bilateral/unilateral enlargement of the anterior chamber of the eye, particularly in the dorso-nasal region (orange arrowheads in **G**–**H**, and **K**). **M**–**P**’ Ocular images of adult wild-type (**M**–**P**) and *foxc1a*^*∆CED2−3*^ mutants (**M**’–**P**’) showing bulging in the nasal part of the anterior chamber of the eye (orange arrowheads in **N**’–**P**’). Panels **N**, **P**, **N**’ and **P**’ show the regions outlined by white boxes in panels **M**, **O**, **M**’ and **O**’ at a higher magnification

Examination of *foxc1a*^*∆CED1−3*^ homozygous embryos identified a completely penetrant ocular phenotype, while heterozygous animals appeared normal. The affected embryos showed an enlargement of the anterior chamber of the eye that was visible at 3-dpf and became more pronounced at later stages (Fig. 3D–F, M). Homozygous embryos also demonstrated reduced blood flow in the caudal region (Additional file 2: Video SV1) and juvenile lethality (100% of fish die by ~ 30-dpf). The fish that survived to 30-dpf showed general edema and variable anterior chamber defects (Fig. 4E–H). The majority of animals displayed an enlargement of the anterior chamber that was most pronounced in the nasal–dorsal part of the eye (Fig. 4G, H), along with deformed and irregularly shaped eyes (Additional file 1: Fig. S3).

The *foxc1a*^*∆CED2−3*^ homozygous embryos carrying a deletion encompassing CED2 and CED3 but not CED1 demonstrated a similar fully penetrant embryonic phenotype: an enlargement of the anterior chamber of the eye at 3-dpf that became more pronounced at 4-dpf (Fig. 3G–I, M) and mildly reduced blood flow in the caudal region (Additional file 3: Video SV2); heterozygous embryos did not show any visible phenotype. However, most embryos recovered at later stages and survived to adulthood, thus showing a milder overall phenotype in comparison with the *foxc1a*^*∆CED1−3*^ line. At 30-dpf, only a small percentage (7%) of the juvenile animals displayed an enlargement of the anterior chamber, general edema and lethality, similar to *foxc1a*^*∆CED1−3*^ fish (Fig. 4I–L), while the majority of homozygotes (93%) appeared normal. However, examination of the surviving *foxc1a*^*∆CED2−3*^ homozygotes at later stages (7-month post-fertilization adults) identified visible ocular defects in 18.75% (3 out of 16) (Fig. 4M’–P’).

With respect to the *foxc1a*^*∆CED1*^ line, neither homozygous nor heterozygous embryos showed any visible phenotype and all embryos survived to adulthood, were fertile and bred normally.

In order to determine the specificity of the observed phenotypes to *foxc1a*, we generated compound heterozygous zebrafish carrying the *foxc1a* knockout allele *mw711* ([26]; from here on referred to as *foxc1a*^*KO*^) with either *∆CED1-3* or *∆CED2-3* in *trans* (*foxc1a*^*KO/ΔCED1−3*^ or *foxc1a*^*KO/ΔCED2−3*^). These fish demonstrated similar enlargements of the anterior chamber as seen in the homozygous lines described above (Additional file 1: Fig. S4), thus supporting a role for the deleted regions in normal *foxc1a* function. Compound heterozygous animals carrying the *mw711* and *∆CED1* alleles, *foxc1a*^*KO/ΔCED1*^, showed no visible phenotype.

### Histological and marker analysis of affected embryos from f***oxc1a***^***∆CED1−3***^ and ***foxc1a***^***∆CED2−3***^ lines

**Fig. 5:**
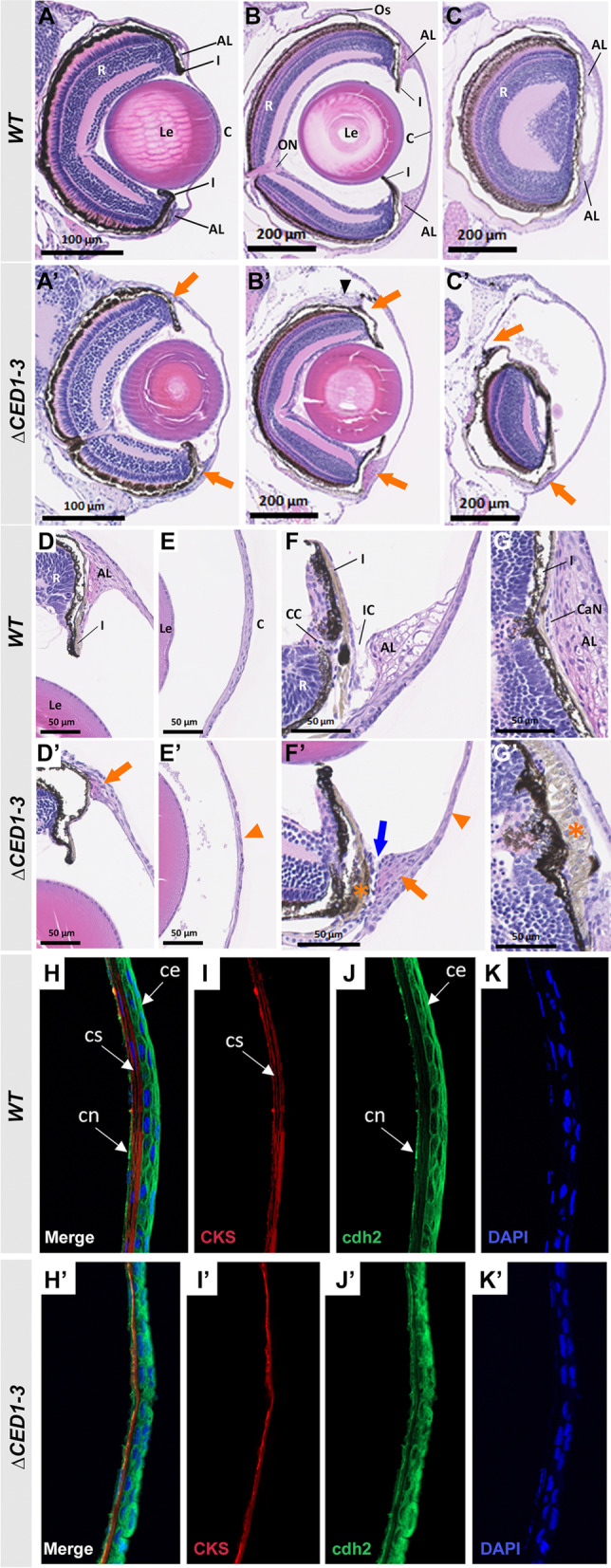
Histological analysis of ocular anomalies in *foxc1a*^*∆CED1−3*^ homozygous embryos. **A**, **A**’ H&E-stained transverse sections of the eye of 6-dpf wild-type and mutant embryos. **B**–**C**’ H&E-stained transverse sections through central (**B** and **B**’) and nasal (**C** and **C**’) eye regions of 30-dpf wild-type and mutant fish. Mutants show a marked enlargement of the anterior chamber and abnormal development of both dorsal and ventral annular ligaments (orange arrows, **A**’–**C**’); dislocation of lenses toward the back of the eye (**B**’); and deformed/misplaced scleral ossicles at the dorsal irido-corneal angle (black arrowhead, **B**’). **D**–**E**’ 20× magnifications of the dorsal irido-corneal angle (**D** and **D**’) and cornea (**E** and **E**’) showing details of the hypoplastic dorsal annular ligament (orange arrow, **D**’), and thin cornea at 30-dpf (orange arrowhead, **E**’). Transverse (**F** and **F**’) and coronal (**G** and **G**’) 40× magnifications of the ventral irido-corneal angle and canalicular network showing an apparent absence of the glycoprotein aggregates in the ventral annular ligament (orange arrow in **F**’), narrowing of the irido-corneal canal (blue arrow, **F**’), hyperplasia of the ventral iris stroma in this region (orange asterisks in **F**’ and **G**’) and thin cornea at 30-dpf (orange arrowhead in **F**’). **H**–**K**’ immunostaining of cornea sections of 30-dpf wild-type and mutant fish with anti-CKS (red) and anti-cdh2 (green), showing a thinner corneal stroma (**I**’) and a disorganized corneal epithelium (**J**’). AL, annular ligament; C, cornea; CaN, canalicular network; CC, ciliary canal; ce, corneal epithelium; cn, corneal endothelium; cs, corneal stroma; I, iris; IC, irido-corneal canal; Le, lens; ON, optic nerve; Os, scleral ossicle R, retina

To further evaluate the developing eye, hematoxylin–eosin (H&E)-stained histological head sections of *foxc1a*^*∆CED1−3*^* and foxc1a*^*∆CED2−3*^ homozygous embryos at 6-dpf were examined. Consistent with the gross morphological observations, an enlargement of the anterior chamber of the eye was noticeable in 6-dpf homozygous embryos from both lines, with *foxc1a*^*∆CED1−3*^ embryos showing a more severe phenotype (Fig. 5A, A’; Additional file 1: Fig. S5A’). In addition to this, variable hypoplasia of the dorsal irido-corneal angle was observed, which again was more pronounced in *foxc1a*^*∆CED1−3*^ embryos. No visible defects in the retina or lens were detected in either line.

Examination of histological transverse and coronal head sections of 30-dpf *foxc1a*^*∆CED1−3*^ mutants identified a considerable enlargement of the anterior chamber with noticeable bulging in the dorsal–nasal area of the cornea being most frequently present (Fig. 5B’–C’; Additional file 4: Video SV3). Additional anomalies included a posteriorly displaced lens with highly reduced/absent vitreous space (Fig. 5B’); absent (4/8) or highly hypoplastic (4/8) dorsal annular ligament (Fig. 5B’, C’) and displaced or deformed scleral ossicles at the dorsal, nasal and temporal irido-corneal angle (Fig. 5B’–D’; Additional file 1: Fig. S5B’, C’; Additional file 4: Video SV3); a thinner cornea (Fig. 5E’); and notable defects in the ventral irido-corneal angle (Fig. 5F’, G’).

Normally, the annular ligament has a fibrous and porous meshwork appearance in aldehyde-fixed preparations (Fig. 5F) and the ‘pores’ were found to be non-membrane-bound aggregates of glycoprotein [29]. In *foxc1a*^*∆CED1−3*^ homozygous embryos, at the ventral annular ligament, a sharp reduction in ‘pores’ was observed, with an overall denser appearance of this tissue (Fig. 5F’). Most importantly, developmental defects in the aqueous humor drainage structure were detected in mutants (Fig. 5F’, G’). Drainage of aqueous humor in zebrafish occurs in a morphologically specialized structure called the canalicular network localized in the ventral irido-corneal angle. In wild-type adult fish, this structure consists of the irido-corneal canal and the ciliary canal that connect the anterior and posterior chambers, respectively, with the angular aqueous plexus where the aqueous humor is returned to the bloodstream [30] (Fig. 5F). The canalicular network is positioned between the iris and the annular ligament and is comprised of endothelial-lined openings of loosely organized juxtacanalicular connective cells; it is functionally analogous to the aqueous humor outflow system in mammals [30] (Fig. 5F). In *foxc1a*^*∆CED1−3*^ mutants, an underdeveloped canalicular network was observed (Fig. 5F’) with hyperplasia of the iris stroma detected in this region in some fish (3/7) (Fig. 5F’, G’). Therefore, the observed enlargement of the anterior chamber could be caused by an increase in the intraocular pressure due to impaired drainage of the aqueous humor.

To further study the cornea defects, 30-dpf *foxc1a*^*∆CED1−3*^ mutant sections were stained for N-cadherin (cdh2) that marks corneal epithelium and endothelium, and corneal keratan sulfate proteoglycan (CKS), which is a marker for corneal stroma [31]. Cornea epithelium at 30-dpf is composed of several layers of epithelial cells, an acellular and well-ordered stroma, and an endothelial monolayer (Fig. 5H–J). Mutant corneas showed thinner stroma and disorganized epithelial layer (Fig. 5I’, J’).

**Fig. 6:**
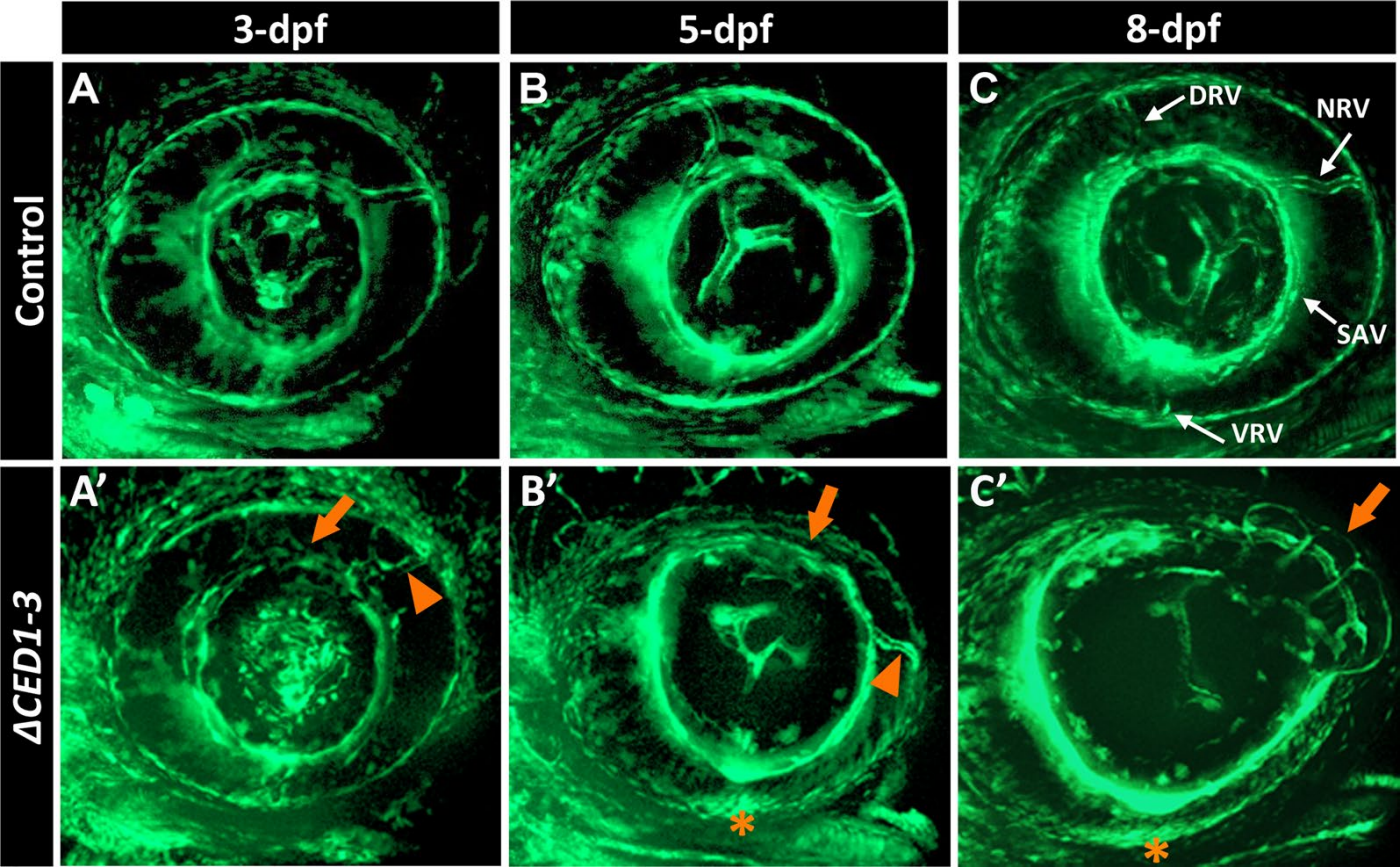
*foxc1a*^*∆CED1−3*^ mutant embryos display defects in the developing superficial choroidal vasculature. **A**–**C**’ Three-dimensional maximum intensity projection images of the ocular vasculature in live control (**A**–**C**) or *foxc1a*^*∆CED1−3*^ homozygous (**A**’–**C**’) embryos carrying *fli1a*:EGFP transgene at 3-, 5- and 8-dpf. Mutant embryos show abnormal development of the dorsal and nasal radial vessels (orange arrowheads in **A**’–**C**’), enlarged and deformed superficial annular vessel (orange arrows) and a highly disorganized vasculogenesis in the ventral part of the eye with no visible ventral radial vessel at 5- and 8-dpf (orange asterisks). DRV (dorsal radial), NRV (nasal radial), SAV (superficial annular), and VRV (ventral radial) blood vessels are indicated

Since *foxc1a*^*KO*^ embryos showed defects in the hyaloid vasculature [26], we studied this structure in the *foxc1a*^*∆CED1−3*^ homozygotes in a Tg(*fli1a*:EGFP) background. Tg(*fli1a*:EGFP) expresses eGFP under the control of the *fli1a* promoter, which is an early endothelial marker that allows monitoring of blood vessel formation [32]. During normal eye development at 3-dpf the superficial choroidal vasculature comprises three radial vessels, nasal (NRV), dorsal (DRV) and ventral (VRV), that project from the periphery of the optic cup toward the lens and are connected by a ring-shaped vessel named the superficial annular vessel (SAV); the same vessels continue to develop at 5-dpf and 8-dpf (Fig. 6A–C) [33]. Examination of 3- and 5-dpf *foxc1a*^*∆CED1−3*^ homozygotes revealed an enlarged SAV and disorganized NRV including irregular shape and/or bifurcation (Fig. 6A’ and B’). At 8-dpf, mutant eyes show a more diffuse *fli1a* signal exposing abnormal development of the superficial choroidal vasculature, a more pronounced enlargement and irregularity (particularly in the dorsal–nasal part) of the SAV (Fig. 6C’), and a misplaced (in all) and erroneously divided into daughter branches (in about half) NRV (9/20). The DRV can be detected in all mutants; however, it had not grown enough to connect to the SAV in most (16/20). Most remarkably, the VRV was not detectable in the ventral part of the eye of all but one mutant (19/20) (Fig. 6C’).

Excavation of the optic nerve head is a recognized clinical feature of glaucoma indicating likely death of retinal ganglion cells. Considering this, we examined the appearance of retinal ganglion cells in 30-dpf juvenile *foxc1a*^*∆CED1−3*^ homozygous mutants in a transgenic Tg(*gap43*:eGFP) background. The Tg(*gap43*:eGFP) line expresses eGFP under the promoter of *gap43*, an axon growth-associated gene, which is expressed during developmental or regenerative axon growth [34] and is a useful tool to monitor optic nerve damage and regeneration [35]. Since retinal axons are still growing at 30-dpf, a similar signal was observed in *foxc1a*^*∆CED1−3*^ and control siblings (Additional file 1: Fig. S6A–F). However, several structural differences in *foxc1a*^*∆CED1−3*^ mutant eyes in comparison with their normal siblings were observed: The distribution of axons was irregular, exposing thinner axon bundles and reduced branching in mutant eyes (Additional file 1: Fig. S6C–F); additionally, the head of the optic nerve appeared to be enlarged in many (5/7) and irregularly shaped (elongated instead of circular) in some (2/7) (Additional file 1: Fig. S6C, E).

Finally, since *foxc1a* is expressed in neural-crest (NC) derived periocular mesenchyme, we examined this cell population in embryos carrying the *foxc1a*^*∆CED1−3*^ allele in a Tg(*foxd3*:GFP)^zf15^ transgenic background (expressing GFP in migrating NC cells [22]). We observed no visible difference in intensity or distribution of GFP-positive cells between control (wild-type or heterozygous) and *foxc1a*^*∆CED1−3*^ homozygous embryos (Additional file 1: Fig. S7), suggesting no defects in migration of NC cells to the periocular mesenchyme in this mutant.

### Analysis of gene expression in lines carrying deletions of downstream elements, foxc1a^***∆CED1−3***^***, foxc1a***^***∆CED2−3***^ and ***foxc1a***^***∆CED1***^

**Fig. 7:**
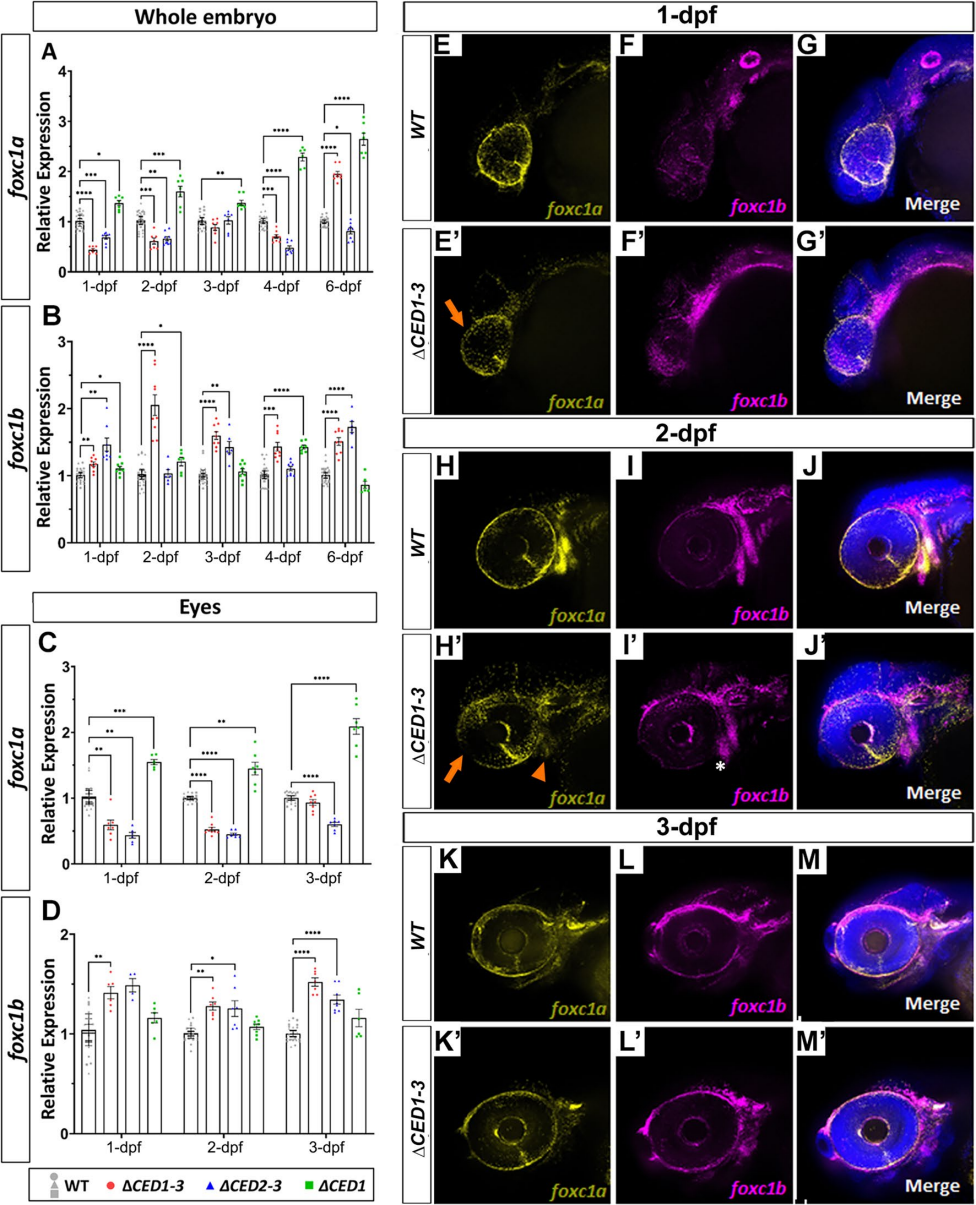
Expression studies in zebrafish mutants carrying deletions of downstream regions of *foxc1a*. qRT-PCR relative expression of *foxc1a* (**A**, **C**), *foxc1b* (**B**, **D**) transcripts in 1–6-dpf whole bodies (**A**, **B**) and 1–3-dpf dissected eyes (**C**, **D**) of wild-type and mutant embryos; *: *p* < 0.05; **: *p* < 0.01; ***: *p* < 0.001; ****: *p* < 0.0001. **E**–**M**’ RNAscope in situ hybridization analysis of *foxc1a* (yellow) and *foxc1b* (magenta) expression in 1-, 2- and 3-dpf wild-type and *foxc1a*^*∆CED1−3*^ mutant embryos. Mutant embryos showed a visible reduction in ocular *foxc1a* expression at 1- and 2-dpf (orange arrows in **E**’ and **H**’) as well as in the branchial arches at 2-dpf (orange arrowhead in **H**’), while expression of *foxc1b* appeared normal (white asterisk; **I**’)

To determine the effect of the downstream deletions on the expression of *foxc1a* and *foxc1b*, qRT-PCR experiments were performed using wild-type and homozygous mutant whole embryo (1-, 2-, 3-, 4- and 6-dpf) and ocular (1-, 2- and 3-dpf) RNA samples. This analysis identified a significant decrease in the *foxc1a* transcript level at early stages in both whole embryos and eyes in *foxc1a*^*∆CED1−3*^ and *foxc1a*^*∆CED2−3*^ mutants (Fig. 7A, C). *foxc1a*^*∆CED1−3*^ homozygotes demonstrated a downregulation of *foxc1a* for both whole embryos (ranging from 0.44- to 0.61-fold) and mutant eyes (ranging from 0.52- to 0.59-fold) at 1–2-dpf and no difference at 3-dpf (Fig. 7A, C). Expression levels in *foxc1a*^*∆CED2−3*^ homozygotes were decreased at most stages in both embryos (ranging from 0.48- to 0.82-fold across all stages with the exception of 3-dpf) and mutant eyes (0.43- to 0.6-fold across all stages) (Fig. 7A, C). On the other hand, *foxc1a*^*∆CED1*^ homozygotes demonstrated a significant upregulation of *foxc1a* at all stages in both whole embryos (ranging from 1.67- to 2.64-fold) and eyes (1.45- to 2.09-fold) (Fig. 7A, C). These results strongly suggest that the downstream regions of *foxc1a* are involved in its transcriptional regulation.

In addition to *foxc1a*, expression of *foxc1b* was also affected in *foxc1a*^*∆CED1−3*^, *foxc1a*^*∆CED2−3*^, and *foxc1a*^*∆CED1*^ mutants. The *foxc1a*^*∆CED1−3*^ embryos showed an increase in the *foxc1b* level at all stages in whole embryos and eyes (Fig. 7B, D). The *foxc1a*^*∆CED2−3*^ embryos similarly demonstrated upregulation in *foxc1b* expression in whole embryos and eyes at some stages (Fig. 7B, D). The *foxc1a*^*∆CED1*^ embryos displayed no difference in *foxc1b* expression in mutant eyes at any stage, while mild effects (upregulation or downregulation) were detected in whole embryos at some stages (Fig. 7B, D).

Whole-mount in situ hybridization for *foxc1a* and *foxc1b* as well as antibody staining for Foxc1 in wild-type and *foxc1a*^*∆CED1−3*^ mutants at 1-, 2- and 3-dpf revealed weaker *foxc1a*/Foxc1 staining in the periocular mesenchyme and the branchial arches at 1- and 2-dpf (Fig. 7E’, H’; Additional file 1: Fig. S8A’–C’), consistent with the qPCR analysis.

## Discussion

Despite the wide use of exome and genome sequencing, a large number of individuals with inherited disorders lack a genetic diagnosis [36, 37]. While there are still novel factors to be discovered, various studies highlight the importance of noncoding regions in human disease [17, 38–40]. One important class of functional sequences located in noncoding regions are regulatory elements that can be predicted based on evolutionary conservation, open chromatin state, the three-dimensional structure of chromatin, and other approaches [20, 27, 41]. Variants in these regions affect gene expression through alteration/removal of binding sites for transcription factors and/or disturbing the three-dimensional structure of chromatin [42, 43]. Consistent with this, many studies have shown that mutations in *cis*-regulatory elements of disease-associated genes can cause similar phenotypes to the ones reported for coding region variants [4, 13, 14, 16].

*FOXC1* encodes a forkhead box transcription factor involved in vertebrate embryonic development. *FOXC1* is located at 6p25.3 in a conserved cluster of *FOX* genes (*FOXQ1*, *FOXF2* and *FOXC1*). Mutations in *FOXC1* are responsible for several developmental disorders of the anterior segment of the eye [3–5, 7, 8, 10], while no human disease phenotypes are currently identified for *FOXF2* or *FOXQ1*. Copy number variants represent an important class of *FOXC1* pathogenic alleles and include both deletions and duplications of this gene [3, 4, 8, 44]. This highlights the importance of a precise dosage of *FOXC1* for proper development. Accordingly, disruption of *FOXC1* regulatory elements, or its upstream factors, is likely to result in disease; however, the mechanisms of *FOXC1* regulation are currently unknown. In this manuscript, we present the first data on *cis*-regulatory elements of *FOXC1* that have been studied in vivo*.*

We identified five conserved elements in the zebrafish *foxc1a* and *foxc1b* genomic environment corresponding to four human noncoding *FOXC1* regions. Two of these elements are situated upstream of *foxc1a* or *foxc1b* and relate to the same remote upstream region of *FOXC1*, while the other three are located downstream of *foxc1a/FOXC1.* The conserved elements ranged from 67 to 160 bp in length and showed 76–84% identity between zebrafish and human. In terms of their position within the conserved block, the distant upstream elements reside 5’ of the *FOXF2/FOXC1* or *foxf2/foxc1* clusters, 27.9-/12.94 kb from *foxc1a/b* and 221.96 kb from *FOXC1*. The downstream elements are located at 2.5/2.9 kb to 151/290 kb downstream of *foxc1a/FOXC1*. In humans, one downstream element is situated in the intergenic region between *FOXC1* and *GMDS* while the other two are located within an intronic region of *GMDS.* In zebrafish, all three downstream elements reside in the intergenic region between *foxc1a* and the neighboring gene *mylk4a*, since *gmds* is bordering *foxc1b* but not the *foxc1a* ortholog; interestingly, despite this downstream synteny for *foxc1b*, no conserved elements were identified in this region. *foxc1a* represents the main zebrafish ortholog of *FOXC1* in terms of functional significance: *foxc1a*^*KO*^ fish display strong developmental defects that recapitulate *FOXC1* disease-associated features, while *foxc1b*^KO^ fish do not show any visible phenotype [26]. The presence of conserved elements downstream of the *foxc1a* gene despite the lack of syntenic *gmds* further supports a possible role for these elements in the regulation of *foxc1a*. This is consistent with evolutionary studies suggesting that when genomic duplications occur, as in zebrafish, coding sequences of the extra bystander gene may be erased, while cis-regulatory modules for developmental genes remain conserved [12, 45].

Deletion of predicted *cis*-regulatory elements in animal models has become a powerful approach to exploring their role in gene regulation and disease [20, 41, 46, 47]. To investigate the possible function of the identified sequences, we generated a series of zebrafish lines carrying various deletions encompassing the identified candidate elements. The deletion of a 152 kb region comprising all three downstream regions (CED1-3) resulted in developmental defects in the anterior segment of the eye and juvenile lethality. Further dissection of this region revealed that deletion of an 82.7 kb fragment containing two of the three downstream conserved elements (CED2-3) produced a similar but milder/transient phenotype, while removal of only the first downstream element (CED1; 69.1 kb deletion) did not have any noticeable effect on zebrafish development or survival. In terms of expression changes, deletions of CED1-3 or CED2-3 resulted in significant downregulation of the *foxc1a* transcript in zebrafish embryos and developing eyes while removal of CED1 caused a detectable increase in the level of *foxc1a* transcript. In contrast, deletions of distant conserved elements upstream of either *foxc1a* (∆CEU1a) or *foxc1b* (∆CEU1b) did not produce a visible phenotype; zebrafish lines lacking the CEU1a or CEU1b elements had a minor change in *foxc1a/b* expression but showed a considerable alteration in the levels of *foxf2a/b* and *foxq1a/b* transcripts located in close proximity to those elements. A complementation test confirmed that the *∆CED1-3* and *∆CED2-3* noncoding deletions and *foxc1a* knockout allele *mw711* [26] are allelic to each other and thus all affect *foxc1a*.

The phenotype observed in *foxc1a*^*∆CED1−3*^ and *foxc1a*^*∆CED2−3*^ mutants is consistent with features reported in *foxc1a*^*KO*^ animals, including ocular abnormalities, vascular/blood flow defects, edema and lethality [26] but shows later onset and milder presentation. The later onset/milder phenotype in mutants with regulatory region deletions is likely related to the higher *foxc1a* level as these animals have at least 40% of normal *foxc1a* in comparison with its complete absence in *foxc1a*^KO^ embryos. Similar phenomena were described for other transcription factors with dosage-dependent phenotypes [20, 41, 46, 47].

Drainage of the aqueous humor in humans takes place through a circular structure located in the irido-corneal angle that includes the trabecular meshwork (TM) and Schlemm’s canal [48]. The TM is a porous structure formed by several layers of connective tissue beams and collagenous elastic fibers. The TM represents the main pathway for drainage of aqueous humor out of the eye with a critical role in maintaining normal intraocular pressure; its function is to provide a pressure gradient resistance to the aqueous humor flow to the Schlemm’s canal [48]. Schlemm’s canal is considered a unique blood–lymphatic intermediate-type vessel that is originally formed by endothelial cells from the choroidal vein and acquires lymphatic characteristics later in development [49]. Unlike in humans, drainage of aqueous humor in zebrafish occurs in the ventral part of the irido-corneal angle only, through the canalicular network and the angular aqueous plexus (homologous structures to the human trabecular meshwork and Schlemm’s canal, respectively) [30]. The *foxc1a*^*∆CED1−3*^ mutants demonstrated defects in the ventral canalicular network, possibly related to the noted abnormalities in the development of the ventral superficial choroidal vasculature of the eye. Thus, the observed enlargement of the anterior chamber (indicating an increase in intraocular pressure) is likely due to an impairment of aqueous humor drainage through the malformed outflow structures in affected animals. High intraocular pressure accompanied by an enlargement of the ocular globe (known as buphthalmos) is a common manifestation of congenital glaucoma [50], one of the developmental phenotypes associated with mutations in *FOXC1*. The mechanism of this disorder is not fully known; however, human studies identified developmental defects in the drainage structures of patients with congenital glaucoma caused by *CYP1B1* mutations [51]. No similar reports are available for *FOXC1*, but studies in mice demonstrated that *Foxc1*^+/-^ [52] heterozygotes as well as animals homozygous for mutations in congenital glaucoma genes *Cyp1b1* [53] and *Angpt* [54] have abnormally formed trabecular meshwork and/or Schlemm’s canal. Thus, the generated *foxc1a* mutants will serve as powerful models for studies of human developmental glaucoma.

Expression levels of the *foxc1a* transcript were altered in all downstream deletion lines implying a regulatory role for those regions. Although the deleted regions comprised large noncoding segments, it is plausible to assume that the identified conserved elements within the region are making the most important contribution to the observed regulatory effect. However, other factors, such as the presence of additional, yet-to-be-identified, regulatory DNA elements and/or noncoding RNA in this region as well as possible positional effects due to the changes in the architecture of topological associating domains (TADs) often associated with larger genomic deletions [55, 56], cannot be ruled out. Further studies including deletions of each downstream element separately could provide further information about their distinct functions.

Interestingly, deletions of *foxf2a-foxc1a* or *foxf2b-foxc1b* intergenic fragments positionally orthologous to the region containing the POAG-associated rs2745572[A] SNP [18] but lacking sequence conservation resulted in downregulation of *foxc1a* or *foxc1b* expression, respectively, indicating a possible role in transcriptional regulation. This is supported by the growing evidence that *cis-*regulatory regions may diverge in their primary sequences in different species while maintaining their functional (regulatory) role [57]. Another possibility is that these intergenic deletions affected distances between other regulatory elements and/or overall chromatin structure which was followed by a negative effect on *foxc1a* and *foxc1b* expression; however, the specific nature of the observed changes (limited to late developmental stages in both zebrafish lines) is consistent with the likely presence of distinct *cis*-regulatory element(s) in these regions. Further dissection of these regions in both human and zebrafish may provide additional insight into the location of regulatory sequences and their roles in *FOXC1/foxc1* expression and POAG.

In summary, this study identified several regulatory regions that are critical for the normal expression of *FOXC1/foxc1* in vertebrates. Specifically, we show that *foxc1a* and thus likely *FOXC1* embryonic expression is governed by conserved elements located downstream of the gene and that deletions of these elements result in a range of phenotypes with variable severity in zebrafish. Further studies of these regions in human patients are likely to explain additional cases of Axenfeld–Rieger syndrome, aniridia, Peters anomaly, and glaucoma, and may possibly contribute to the extreme variability in phenotypes caused by *FOXC1* heterozygous variants.

## Materials and methods

### Analysis of sequence conservation at the nucleotide level

Visual analysis of the Vertebrate Multiz Alignment & Conservation (100 Species) track at UCSC Genome Browser (http://genome.ucsc.edu) included a region from the start of human chromosome 6 (~ 1.6-Mb upstream *FOXC1*) until the start of *MYLK* (~ 1-Mb downstream *FOXC1*)*.* Regions of high conservation from human to zebrafish (excluding coding regions) were selected and alignments were verified. Additionally, BLAST comparisons of *FOXC1/foxc1a/foxc1b* genomic regions were carried out manually: relevant human (hg38) or zebrafish (DanRer11) genome sequences were aligned with the RefSeq zebrafish or human genome, correspondingly, using BLASTN and the ‘somewhat similar’ alignment option (coding regions, UTRs and immediate promoters were disregarded).

### Animal husbandry

Zebrafish (Danio Rerio) were raised and maintained under standard conditions as previously described [31]. The Tg(*fli1a*:eGFP), Tg(*gap43*:eGFP) and Tg(*foxd3*:GFP) lines were used to monitor blood vessel [32], optic nerve development [34] and neural-crest populations [58]. Developmental stages were determined by previously described morphological criteria [59]. All experiments were conducted in accordance with the guidelines established by the Institutional Animal Care and Use Committee at the Medical College of Wisconsin.

### Generation of genomic deletions in zebrafish

Integrated DNA Technologies (IDT, Coralville, IA) custom Alt-R CRISPR-Cas9 guide RNA tool (https://us.idtdna.com/site/order/designtool/index/CRISPR_CUSTOM) was used to design sgRNAs. Two guides were designed for each deletion, one at each flank (Additional file 1: Table S2). Trans-activating CRISPR RNA (tracrRNA), Cas9 and sgRNAs were purchased from IDT. Each sgRNAs (7.5 μM) and tracrRNA (7.5 μM) were mixed, incubated for 5 min at 95ºC and cooled at room temperature. Cas9 protein (Alt-R® CRISPR-Cas9, IDT) and the pair of tracrRNA/sgRNA complexes were mixed to a final concentration of 0.5 μg/μL for Cas-9 and 1.5 μM for each complex and incubated for 10 min at 37 °C. Single-cell-stage embryos were microinjected with 9.2nL of Cas9/tracrRNA/sgRNA complex and 0.05% Phenol red (Sigma, St. Louis, MO) using the Nanoject II Injector (Drummond Scientific, Broomall, PA). Mosaic breeders were identified by analysis of their offspring via PCR amplification and sequencing with specific primers to identify mutant and wild-type alleles (Additional file 1: Table S3). Founder fish carrying deletions (Additional file 1: Table S1) were selected for further analysis and corresponding lines were established.

### Morphologic analysis of embryos and adults

Zeiss SteREO Discovery V12 microscope (Carl Zeiss, Thornwood, NY) with either a 1.0X stereo objective or a 10X compound objective and Nikon SMZ-1500 with a 1.0X stereo objective were used for gross morphological observations and imaging. Fluorescent maximum intensity projection of z-stack images of transgenic line embryos was obtained using an AxioImager.Z1 microscope with an ApoTome attachment, an AxioCam 503 mono camera and ZEN pro software (Zeiss). Anterior chamber surfaces were measured using dorsal images and ImageJ 1.52 k [60]. Student’s t test was used to determine statistical significance.

### Histological studies

Adult fish and embryos were immersed overnight in modified Davidson’s fixative (30% of a 37% solution of formaldehyde, 15% ethanol, 5% glacial acetic acid, and 50% distilled H_2_O) [61] and then transferred to 70% ethanol. Fixed samples were submitted to the Children’s Research Institute Histology Core at the Medical College of Wisconsin for paraffin sectioning and hematoxylin–eosin staining per standard protocols. NanoZoomer digital slide scanner was used to image the slides and NDP.view2 viewing software was used to visualize the images (Hamamatsu, Hamamatsu City, Japan).

### *RNAscope *in situ* hybridization and immunofluorescence*

Whole embryos were fixed overnight in 4% paraformaldehyde and then transferred to 100% methanol. Embryos were hybridized with RNAscope probes for *foxc1a* (499611-C2), and *foxc1b* (584981-C3) (Advanced Cell Diagnostics, Newark, CA) using manufacturer protocols with minor modifications [31].

For immunohistochemistry, whole-mount embryos or paraffin sections were stained with DAPI (62247; Thermo Fisher, Waltham, MA) and various antibodies including human anti-FOXC1 (8758; Cell Signaling, Danvers, Ma), anti-cdh2 (GTX125962, GeneTex, Irvine, CA) and anti-CKS (MAB2022, Millipore, Burlington, MA) primary antibody, as well as donkey anti-rabbit Alexa Fluor 488 (A21206, Thermo Fisher) and donkey anti-mouse Alexa Fluor 568 (A10037, Thermo Fisher) secondary antibody, as previously described [31].

### Quantitative RT-PCR transcript level analysis of wild-type and mutant

RNA extraction of whole embryos or dissected eyes was performed using Direct-zol RNA MiniPrep (Zymo Research, Irvine, CA); all samples were treated with DNase I (Invitrogen) prior to cDNA synthesis.

cDNA was synthesized using SuperScript III reverse transcriptase (Thermo Fisher). CFX384 Touch Real-Time PCR Detection Systems (BioRad, Hercules, CA), SYBR Green PCR Master Mix (Applied Biosystems) and transcript-specific primers (Additional file 1: Table S4) were used to analyze selected genes by real-time qPCR. β-actin (*actb1*) was used as the reference gene for the relative quantification of expression levels. All samples were run in triplicate to obtain average Cq values. Technical replicates that fell multiple standard deviations from the average were considered outliers and, in agreement with standard practice, removed from the analysis. Total fold changes and standard deviations were calculated as the average of three independent biological repeats via the 2^−ΔΔCt^ method [62]. Student’s t test was used to determine statistical significance.

## Supplementary Information

**Additional file 1.** Supplemental material including Supplemental Figures S1 to S8 and their legends, Supplemental Tables S1 to S4 and Supplemental Videos legends.

**Additional file 2.** Supplemental Video SV1. In vivo imaging of blood flow in wild-type and *foxc1a*^*ΔCED1-3*^ homozygous embryos at 78-hpf.

**Additional file 3.** Supplemental Video SV2. In vivo imaging of blood flow in wild-type and *foxc1a*^*ΔCED2-3*^ homozygous embryos at 78-hpf.

**Additional file 4.** Supplemental Video SV3. Transverse serial sections of 1-mpf wild-type and *foxc1a*^*ΔCED1-3*^ homozygous embryos.
