# Supplementary material for: CRISPR-Cas9-mediated functional dissection of the foxc1 genomic region in zebrafish identifies critical conserved cis-regulatory elements
Source: Hum Genomics. 2022 Oct 25;16:49. doi: 10.1186/s40246-022-00423-x (PMC9597995; doi:10.1186/s40246-022-00423-x)
Supplement: Supplementary file 1 — Additional file 1. Supplemental material including Supplemental Figures S1 to S8 and their legends, Supplemental Tables S1 to S4 and Supplemental Videos legends. [file 40246_2022_423_MOESM1_ESM.pdf]

SUPPLEMENTAL MATERIAL

CRISPR-Cas9-mediated functional dissection of the *foxc1* genomic region in zebrafish identifies critical conserved *cis*-regulatory elements

Jesús-Jose. Ferre-Fernández , Sanaa Muheisen, Samuel Thompson, Elena V. Semina.

- **Supplemental Figures S1 to S8 and their legends**
- **Supplemental Tables S1 to S4**
- **Supplemental Videos legends**

Ferre-Fernandez_Supp._Fig._S1

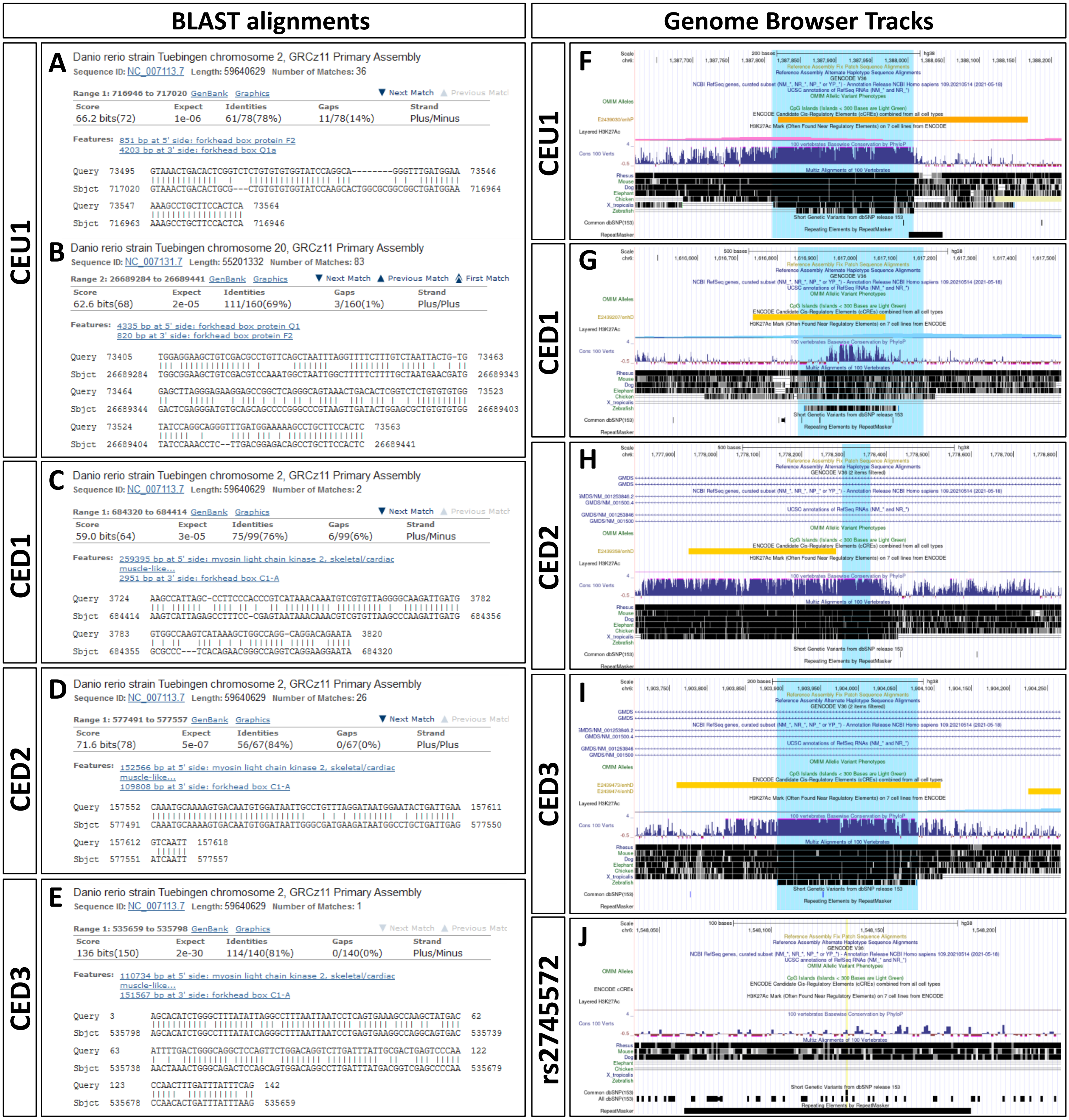

Supplemental Figure S1. Summary information for candidate regulatory regions in the human *FOXC1* genomic region. (A-E) BLAST alignments of human and zebrafish sequences for each of the four conserved elements CEU1, CED1, CED2 and CED3. (F-I) Screenshots of the Genome Browser showing the location and publicly available information for each of the four conserved elements; blue highlight indicates the position of each conserved element; conservation/regulatory tracks include Phylop score of conservation of 100 vertebrates, Multiz alignment of 100 vertebrates, and ENCODE Candidate Cis-Regulatory Elements (cCREs). Please note that the four elements are located in highly conserved regions and overlap or border candidate ENCODE cCRES. (J) Screenshot of the Genome Browser showing the position (yellow highlight) of the SNP rs2745572[A] in the human *FOXC1* region; no sequence conservation or cCREs were identified in this region in zebrafish.

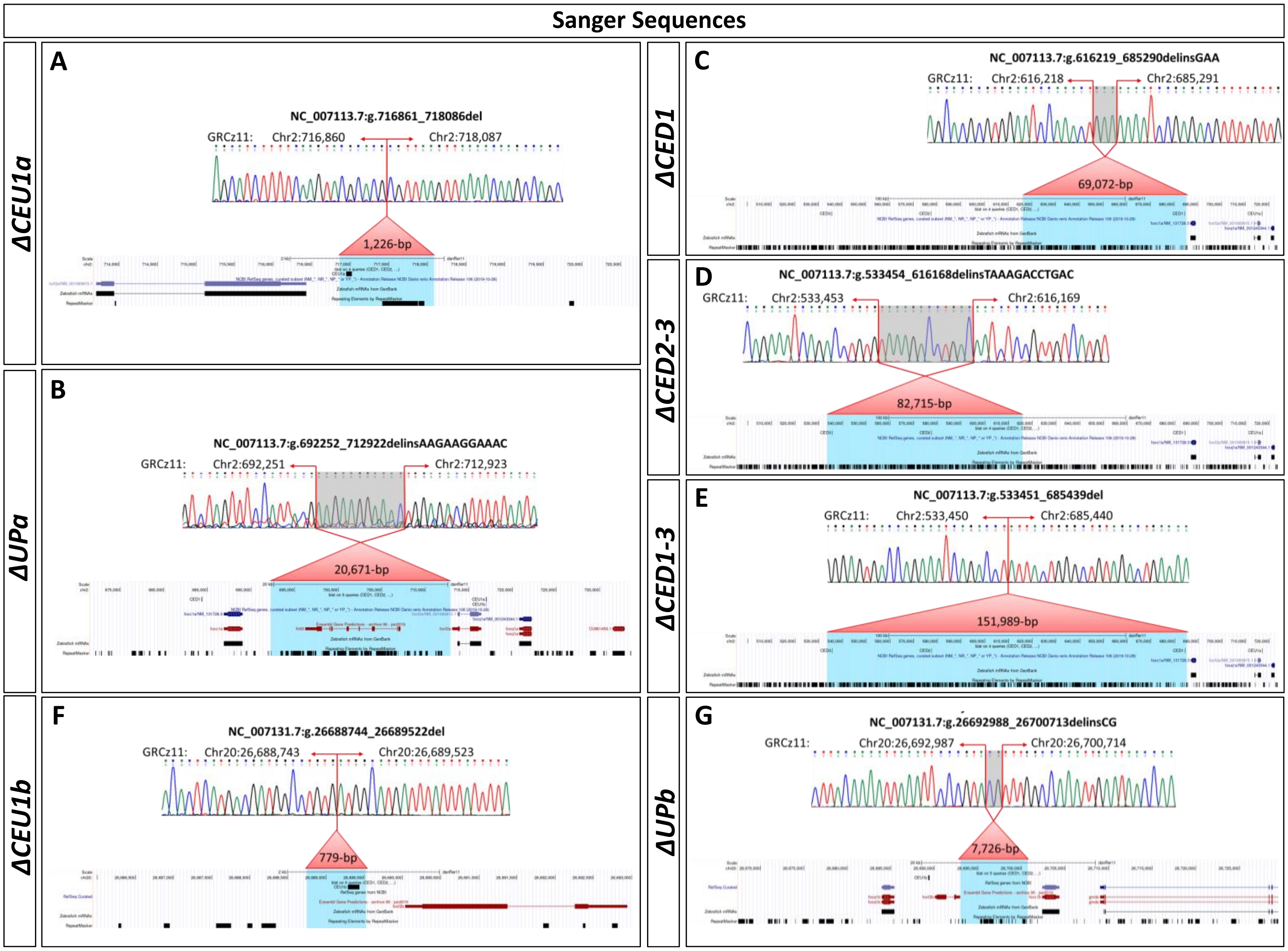

Supplemental Figure S2. Schematics and Sanger chromatogram traces for deleted regions in zebrafish. (A-G) Sanger DNA chromatograms of the seven non-coding deletions generated in this study and schematic drawing showing their positions in the zebrafish genome. Blue highlight in the Genome Browser image indicates the span of each deletion. Conserved elements are indicated as black boxes in the upper track of the Genome Browser image. RefSeq curated zebrafish genes track and Ensembl gene predictions track are also shown; short erroneous insertions identified at the deletion site of some alleles are shown with grey highlight in the corresponding chromatograms.

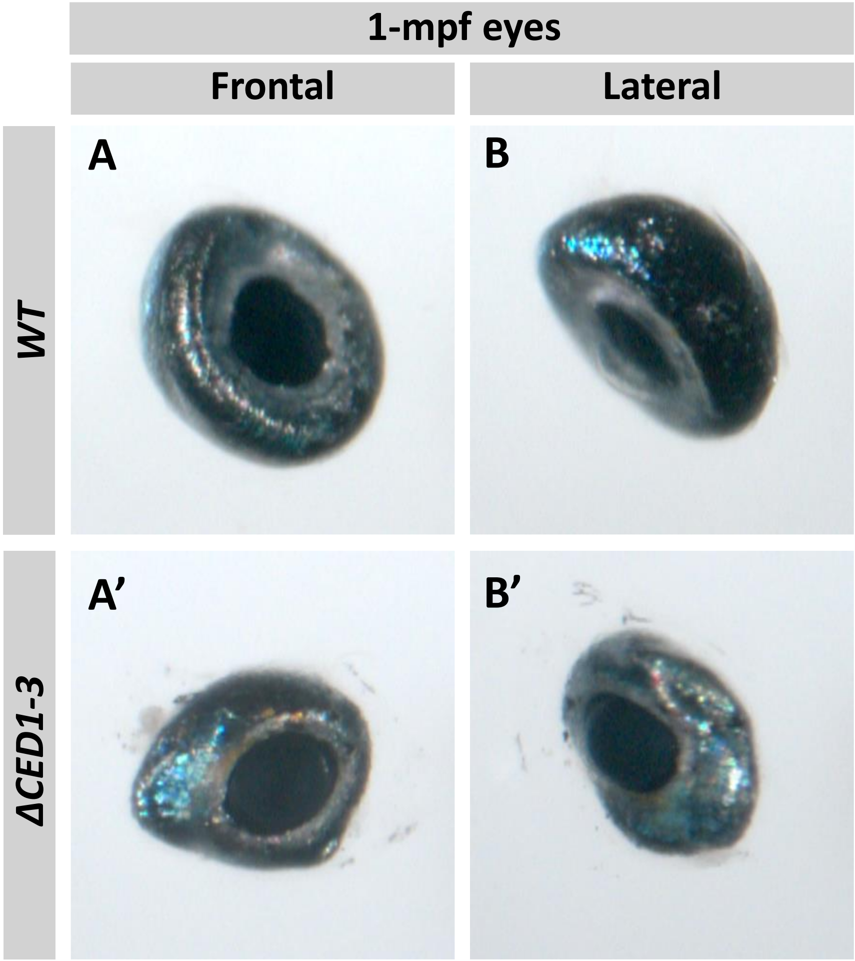

Supplemental Figure S3. The enucleated eyes of paraformaldehyde-fixed 1-mpf *foxc1a*$^{\Delta CED1\text{-}3}$. Wild-type (A, B) and *foxc1a*$^{\Delta CED1\text{-}3}$ mutant (A', B') eyeballs at 1-mpf (months post fertilization) are shown. Please note smaller size and irregular shape of mutant eyeballs.

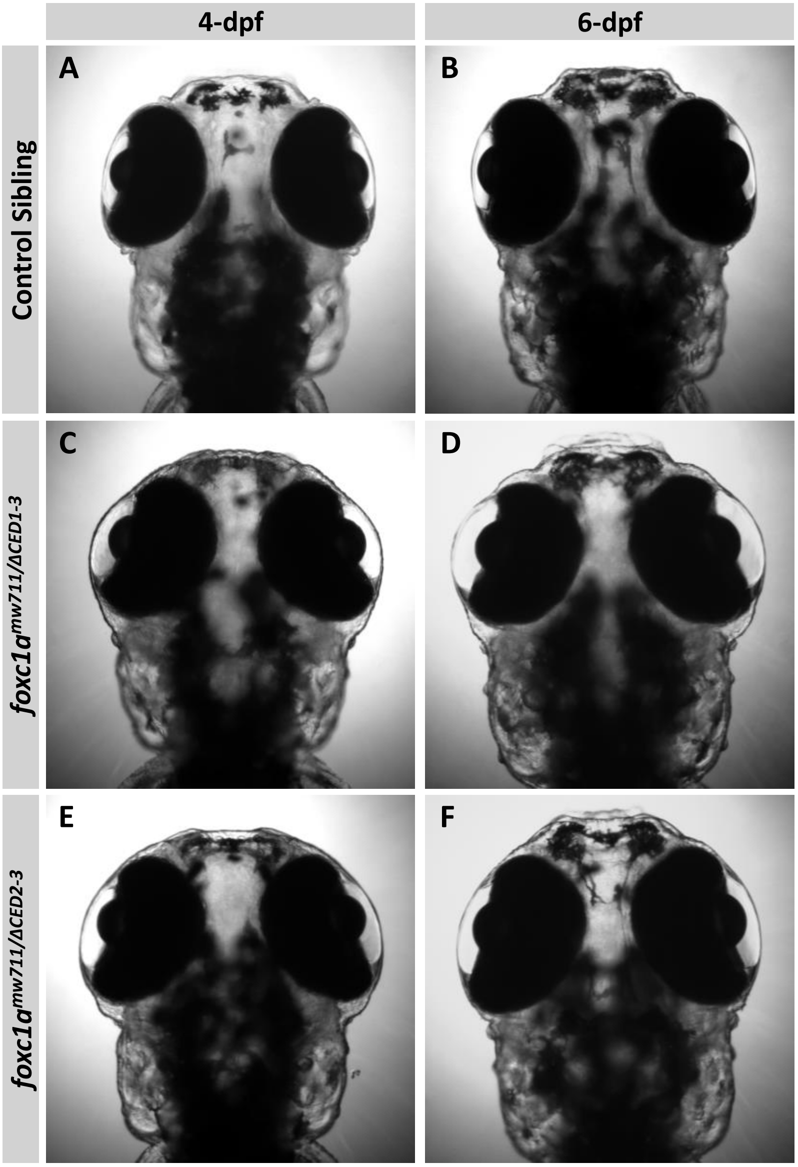

Supplemental Figure S4. Images of *foxc1a*$^{mw711/\Delta CED1-3}$ and *foxc1a*$^{mw711/\Delta CED2-3}$ compound heterozygous embryos. Dorsal head images of 4- and 6-dpf control (A and B) as well as *foxc1a*$^{mw711/\Delta CED1-3}$ (C and D) and *foxc1a*$^{mw711/\Delta CED2-3}$ (E and F) compound heterozygous embryos. Combining the *foxc1a* knockout allele *mw711* with either *ΔCED1-3* or *ΔCED2-3* non-coding deletion alleles results in enlargement of the anterior chamber, similar to the phenotypes observed in homozygous *foxc1a*$^{\Delta CED1-3}$ and *foxc1a*$^{\Delta CED2-3}$ mutants. An unaffected sibling of *foxc1a*$^{mw711/\Delta CED1-3}$ was used as control.

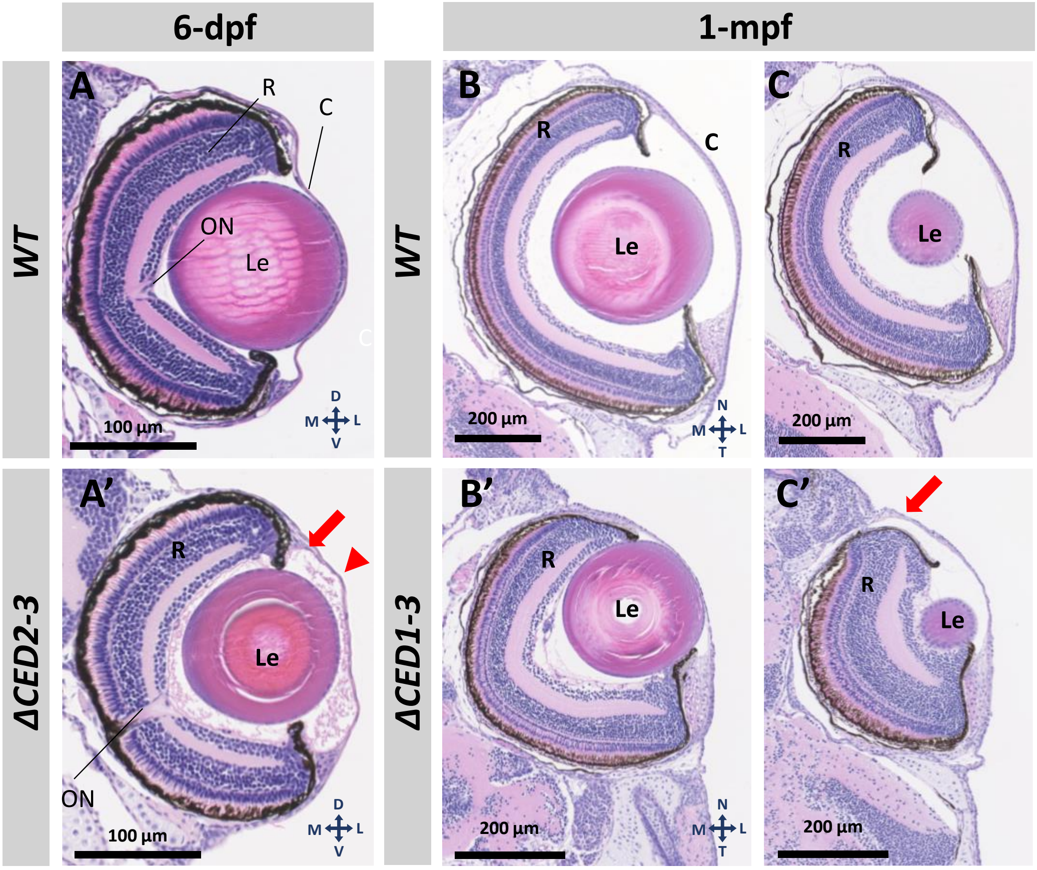

Supplemental Figure S5. Histological analysis of ocular anomalies in *foxc1a*$^{\Delta CED2\text{-}3}$ homozygous embryos and *foxc1a*$^{\Delta CED1\text{-}3}$ homozygous juvenile fish. (A, A') 20X H&E-stained histology transverse sections of the eye at 6-dpf (days post fertilization) wild-type and *foxc1a*$^{\Delta CED2\text{-}3}$ mutant embryos. Please note enlargement of the anterior chamber of the eye (red arrowheads) and hypoplasia of the dorsal (red arrow) irido-corneal angle. No visible structural defects were detected in other parts of the eye. (B-C') 10X H&E-stained central (B and B') and dorsal coronal (C and C') eye sections of 1-mpf (months post fertilization) wild-type and *foxc1a*$^{\Delta CED1\text{-}3}$ homozygous fish. Mutants showed a strong hypoplasia/absence of the nasal annular ligament (red arrow). C, cornea; Le, lens; ON, optic nerve; R, retina. Compass key: D, dorsal; L, lateral; M, medial; N, nasal; T, temporal; V, ventral.

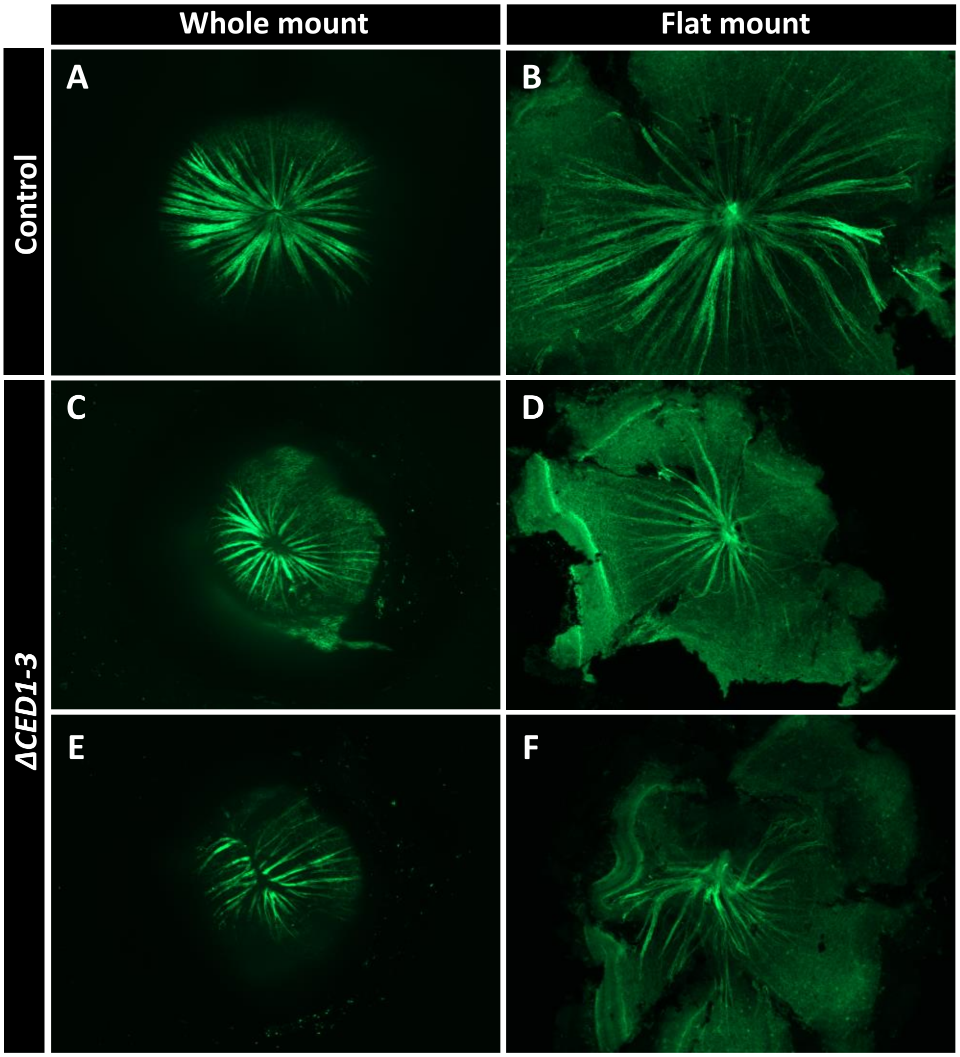

Supplemental Figure S6. Images of the optic nerve head and retinal axons in control and *foxc1a*$^{\Delta CED1\text{-}3}$ homozygous fish. (A-F) Three-dimensional maximum intensity projection images of the retinal axons in whole mount (A, C and E) and flat mount (B, D and F) retinas of fixed 1-mpf (months post fertilization) control (A and B) and *foxc1a*$^{\Delta CED1\text{-}3}$ mutant (C-F) embryos carrying the *gap43*:EGFP transgene. Mutant embryos showed abnormal appearance of the axon bundles with reduced branching (C-F) and enlarged (C) or irregularly shaped (E) optic nerve heads.

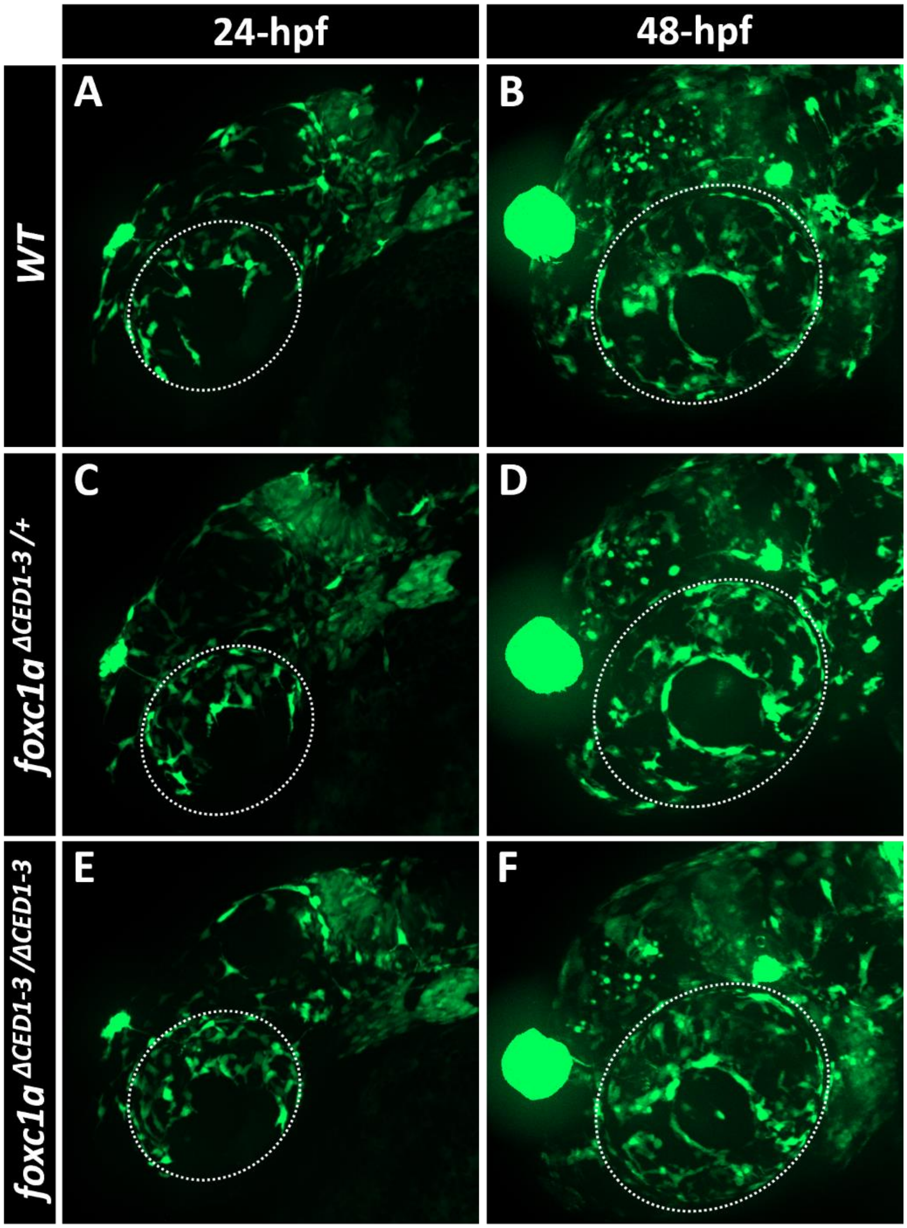

Supplemental Figure S7. Migration of neural-crest derived cells into the periocular mesenchyme is not altered in *foxc1a*$^{\Delta CED1\text{-}3}$ mutants. (A-F) Three-dimensional maximum intensity projection images of neural-crest derived cells (NCC) migrating into the periocular mesenchyme of 24- and 48-hpf (hours post fertilization) wild-type (A, B), *foxc1a*$^{\Delta CED1\text{-}3}$ heterozygous (C, D) and homozygous (E, F) embryos carrying *foxd3*:GFP transgene. Note that controls (A-D) and mutant (E, F) embryos show similar NCC migration patterns (the white dashed lines indicate the eyecup).

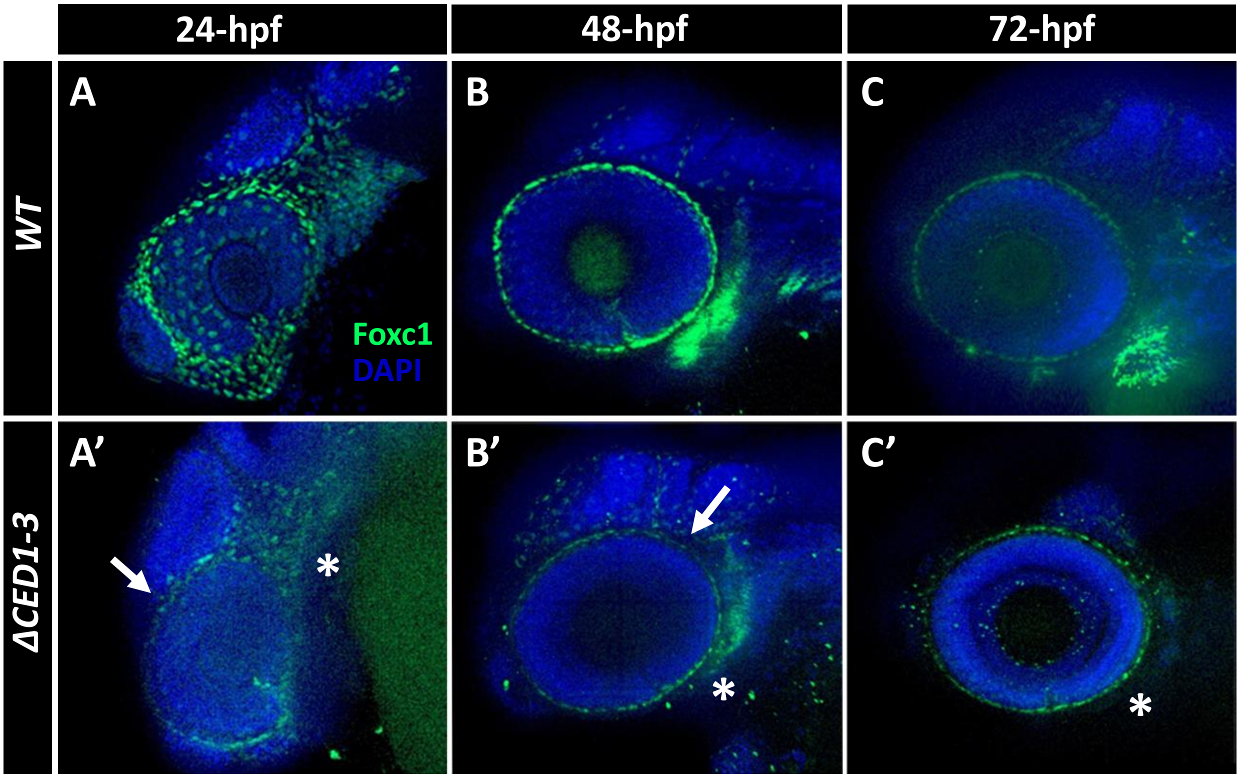

Supplemental Figure S8. Immunohistochemistry with FOXC1 antibody in *foxc1a*$^{\Delta CED1\text{-}3}$ homozygous fish. (A-C') Whole mount staining of 24-, 48- and 72-hpf (hours post fertilization) wild-type (A-C) and *foxc1a*$^{\Delta CED1\text{-}3}$ (A'-C') embryos using rabbit anti-human FOXC1 primary antibody (and Alexa-Fluor 488 conjugated donkey anti-rabbit secondary antibody) that likely recognizes both Foxc1a and Foxc1b proteins due to their high identity. Mutant embryos show a decrease in Foxc1 protein expression in the periocular mesenchyme at 24- and 48-hpf (white arrows) as well as in the branchial arches (white asterisks).

Supplemental Table S1. Coordinates and sizes of *foxc1a/b* non-coding deletions generated in this study.

Deletion	Chromosome	Mutation obtained (GRCzZ11)	size of the deletion (bp)
ΔCEU1a	2	NC_007113.7:g.716861_718086del	1,226
ΔUPa	2	NC_007113.7:g.692252_712922delinsAAGAAGGAAAC	20,671
ΔCED1	2	NC_007113.7:g.616219_685290delinsGAA	69,072
ΔCED2-3	2	NC_007113.7:g.533454_616168delinsTAAAGACCTGAC	82,715
ΔCED1-3	2	NC_007113.7:g.533451_685439del	151,989
ΔCEU1b	20	NC_007131.7:g.26688744_26689522del	779
ΔUPb	20	NC_007131.7:g.26692988_26700713delinsCG	7,726

Supplemental Table S2. Paired gRNAs that were used to produce corresponding genomic deletions at zebrafish *foxc1a/b* loci.

	**CRIPRs guides**	
**Deletion**	**5' guide**	**3' guide**
ΔCEU1a	TTTTCAAGTACGCACGATGG	TAACCTTTTCTGGTGATCAG
ΔUPa	ATGGAAACTAGACCAACTCC	AACGTCTCCACATTGAGAAG
ΔCED1	GCGTGGAATCCAAACTAGAG	TTCGCCACATACCATACCGA
ΔCED2-3	CTCATGGAAAAGTCTCCCAC	ACATGACAACGGAGATTCCA
ΔCED1-3	CTCATGGAAAAGTCTCCCAC	TAATAGGCCCAAAGACCTCG
ΔCEU1b	CTGCGCAAGACTTTGAACAC	TCACTGTCTCCCTCATTTGA
ΔUPb	AATACGATGAAACGGGATTA	TATTCAATTTGGCCAAATAC

Supplemental Table S3. Oligonucleotides for genotyping of wild-type and deletions alleles in corresponding lines.

		Genotyping oligos		
**Deletion**	**Allele**	**Forward**	**Reverse**	**PCR product size**
ΔCEU1a	WT	GACAACGCGCGAACTAAGAG	GCGCACTTTTGCAATTTGATGT	404-bp
	Deletion allele	GCCCCCTAGATCCCCACTAA	GATGTGAGGCGGCTTGTTTC	1019-bp
ΔUPa	WT	GCTATTTCAACCCAAATTAGCCT	CTCAGTCTATGGGGATGGCA	563-bp
	Deletion allele	GCTATTTCAACCCAAATTAGCCT	TTGCACTTTTGTCGGCTTGA	358-bp
ΔCED1	WT	AAACTGACTGCCTGAGTGGG	ACGTCGTGTTAAGCCCAAGA	247-bp
	Deletion allele	GCTGGACCACAGTGAGAACA	GCCCTGTTTAGACCTGAGGG	552-bp
ΔCED2-3	WT	GGGGGAGCACAACTCTACTG	GCAGGCCATTATCTTCATCGC	510-bp
	Deletion allele	CCATCGACACTATGCTGGGA	GGATGTATTTGACACTCTACAGAAG	628-bp
ΔCED1-3	WT	GGGGGAGCACAACTCTACTG	GCAGGCCATTATCTTCATCGC	510-bp
	Deletion allele	CCATCGACACTATGCTGGGA	GCCCTGTTTAGACCTGAGGG	332-bp
ΔCEU1b	WT	TCATCTGACAGGTCACCGAGT	ATGACTTTGGCTCGAGTGGA	335-bp
	Deletion allele	TGCGTATGCGTTAGACCATGA	AGACTGAGTTCGTGCAGCTC	685-bp
ΔUPb	WT	GAAATGCTGTGGCAGTTGTCTT	TCTTTCACATTGGGAGACATTGA	502-bp
	Deletion allele	GAAATGCTGTGGCAGTTGTCTT	GGCAGTTCAGCAGTAAGACATGA	262-bp

Supplemental Table S4. Gene-specific oligonucleotides used for quantitative RT-PCR.

qPCR oligos			
**Transcript**	**Forward**	**Reverse**	**PCR product size**
actb1	GAGAAGATCTGGCATCACAC	ATCAGGTAGTCTGTCAGGTC	323-bp
foxc1a	CGCTATTCCGTCTCCAGTCC	CGCGTGAGAGTACATGGTCA	132-bp
foxc1b	GGCTCGGTCTCTCTGACCTA	CGCTTGCATGTTGCAGTGAT	96-bp
foxf2a	ACACTCCAGTATGCCTCCGT	TGTCACAGACTGGCGATGAG	119-bp
foxq1a	CGGAGTGTTTCGCAGAAGGA	AACTTTCCGCTCGAAGGAGG	126-bp
foxf2b	CCAGCATCCCGGCATATTCA	GTCCTTCCGTTCTCCGACTG	133-bp
foxq1b	GCCATACGCGATTCCAACAC	GTACAGAGTTTCGCCACCCC	112-bp

Supplemental Video SV1. *In vivo* imaging of blood flow in wild-type and *foxc1a*$^{\Delta CED1\text{-}3}$ homozygous embryos at 78 hpf (hours post fertilization). Please note a visibly reduced blood flow in the caudal region of the mutant embryo (red arrow).

Supplemental Video SV2. *In vivo* imaging of blood flow in wild-type and *foxc1a*$^{\Delta CED2\text{-}3}$ homozygous embryos at 78-hpf. Please note a mildly reduced blood flow in the caudal region of the mutant embryo (red arrow).

Supplemental Video SV3. Transverse serial sections of 1-mpf (months post fertilization) wild-type and *foxc1a*$^{\Delta CED1\text{-}3}$ homozygous embryos reveal defects in the anterior segment of the eye.
